# Novel role for conceptus signals in mRNA expression regulation by DNA methylation in porcine endometrium during early pregnancy^†^

**DOI:** 10.1093/biolre/ioac193

**Published:** 2022-11-02

**Authors:** Piotr Kaczynski, Vera van der Weijden, Ewelina Goryszewska-Szczurek, Monika Baryla, Susanne E Ulbrich, Agnieszka Waclawik

**Affiliations:** Institute of Animal Reproduction and Food Research, Polish Academy of Sciences, Olsztyn, Poland; ETH Zurich, Animal Physiology, Institute of Agricultural Sciences, Zurich, Switzerland; Institute of Animal Reproduction and Food Research, Polish Academy of Sciences, Olsztyn, Poland; Institute of Animal Reproduction and Food Research, Polish Academy of Sciences, Olsztyn, Poland; ETH Zurich, Animal Physiology, Institute of Agricultural Sciences, Zurich, Switzerland; Institute of Animal Reproduction and Food Research, Polish Academy of Sciences, Olsztyn, Poland

**Keywords:** early pregnancy, endometrium, pig, prostaglandin E2, estradiol-17β, DNA methylation

## Abstract

During early pregnancy, porcine conceptuses (the embryos with associated membranes) secrete estradiol-17β (E_2_)—their major signal for maternal recognition of pregnancy—and prostaglandin E_2_ (PGE_2_). Both hormones induce prominent changes of the endometrial transcriptome in vivo. Studies on endometrial pathologies have shown that E_2_ affects gene expression by epigenetic mechanisms related to DNA methylation. Herein, we determined the effects of E_2_ and PGE_2_ alone, and a combined E_2_ + PGE_2_ treatment administered into the uterine lumen in vivo on the expression and activity of DNA-methyltransferases (DNMTs) and on CpG methylation patterns of selected genes in porcine endometrium. To compare the effect of treatment with the physiological effect of pregnancy, endometria from day 12 pregnant/cyclic gilts were included. Both E_2_ and PGE_2_ significantly reduced the expression of DNMTs. Likewise, the expressions of DNMT1 and DNMT3A were decreased on day 12 of pregnancy compared to the estrous cycle. DNMT activity increased in endometrial samples following E_2_ treatment and in gilts on day 12 of pregnancy. Treatment with E_2_ alone and/or simultaneously with PGE_2_ altered endometrial DNA methylation of CpG sites of *ADAMTS20*, *ADH1C*, *BGN*, *PSAT1*, and *WNT5A*. Different CpG methylation patterns of *ADAMTS20*, *BGN*, *DMBT1*, *RASSF1*, and *WNT5A* were found in the endometrium on day 12 of pregnancy compared to day 12 of the estrous cycle. Significant correlations were detected between CpG methylation and gene expression for *ADAMTS20*, *ADH1C*, *BGN*, *DMBT1*, *PSAT1*, and *WNT5A*. Our results indicate that CpG methylation induced by embryonic signals may contribute to regulating endometrial gene expression during pregnancy establishment.

## Teaser

Estradiol-17β acting alone and/or simultaneously with PGE_2_ in vivo alters DNA methylation and regulates the expression of genes in the porcine endometrium during early pregnancy.

## Graphical Abstract

**Graphical Abstract:**
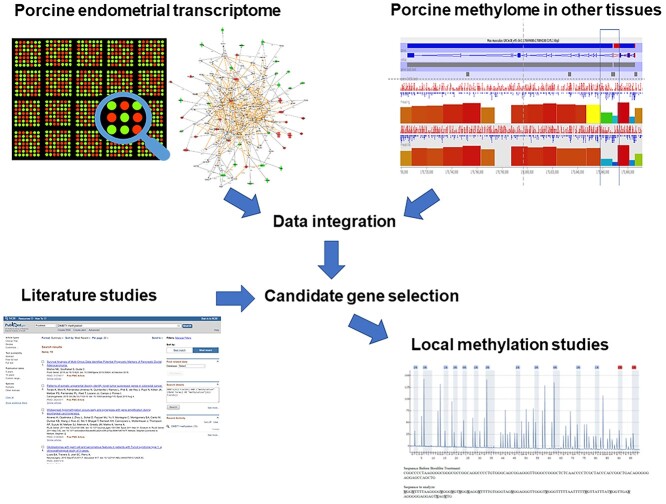

## Introduction

The peri-implantation period in humans and other mammals is a critical stage for pregnancy establishment and development. During this time, the highest mortality rate of embryos is observed and can reach 30% [1]. The reasons for implantation failure may be associated with the embryo’s quality (e.g., genetic disorders or insufficient signaling) as well as with an impaired response of the maternal organism such as disturbed endometrial function or poor uterine receptivity. Many processes, including tissue remodeling, angiogenesis, regulation of immune response, epithelial cell differentiation, cell proliferation, apoptosis, and others, occur in the receptive endometrium and involve a vast number of factors associated with multiple interaction networks [2–5]. Maternal recognition of pregnancy in pigs occurs between days 10 and 13 after fertilization, whereas implantation of embryos takes place between days 14 and 19 of pregnancy (reviewed in [6]). During the process of maternal recognition of pregnancy, the conceptuses, namely the embryos with associated membranes, signal their presence to the maternal organism by secreting multiple factors (steroids, prostaglandins, cytokines and others), allowing for maintained progesterone (P4) secretion by the ovaries and for preparation of the endometrium for embryonic implantation (reviewed in [7]). At the time of maternal recognition of pregnancy, porcine conceptuses secrete increased levels of estrogens, mainly estradiol-17β (E_2_) on days 11–13. Between days 15 and 25–30 after fertilization [8, 9], a second wave of E_2_ secretion occurs. High synthesis and secretion of E_2_ by conceptuses during the maternal recognition of pregnancy coincide with elevated expression of the estrogen receptor (ER) in the endometrial luminal and glandular epithelium [10] and in the conceptus [11], suggesting both autocrine and paracrine actions of E_2_.

The mechanisms of E_2_-dependent regulation of gene expression involve binding of E_2_ to the ligand binding domain (E-domain) of the intracellular ERs, ESR1 and ESR2, resulting in homo- and heterodimer formation by these receptors [12]. Dimerized ERs, as ligand-activated transcription factors, interact with the estrogen-responsive elements (EREs) localized in the DNA sequences of target genes, leading to induced or repressed target gene transcription [13]. Rapid effects observed in response to estrogen treatment are mediated by non-genomic pathways of estrogen signaling [14]. Interestingly, recent studies indicate that genomic and non-genomic effects of E_2_ acting through its receptors are not the only mechanisms by which E_2_ regulates the expression of particular genes. Studies on the mechanisms involved in the etiology of tumors and pathologies of the endometrium have revealed the significant role of E_2_ in epigenetic processes. These were found to be related to DNA methylation and histone modifications, resulting in altered gene expression and development of endometrial pathologies [15–18]. Methylation of DNA is a process by which methyl groups are added to the DNA molecule, resulting in altered gene expression. This process is catalyzed by DNA methyltransferases, including DNMT3A and DNMT3B, which are responsible for establishing new methylation patterns, and DNMT1, which is responsible for the maintenance of inherited methylation patterns [19, 20]. Changes in DNA methylation are involved in the etiology of endometrial cancer and endometriosis [15, 18]. Remodeling of the endometrial tissue during the estrous cycle and pregnancy, and changes associated with developing embryos involve processes that also occur in tumorigenesis, such as vascularization, cell proliferation, and migration. Given the shared molecular mechanisms, it can be assumed that the E_2_-induced epigenetic mechanisms responsible for tumor development may likewise be involved in the physiological regulation of endometrial function. Moreover, it has been shown that DNA methylation changes in the endometrium were correlated with gene expression changes during the transition from the pre-receptive to receptive phase in humans [21, 22]. Therefore, we hypothesize that the processes accompanying implantation and development of pregnancy are regulated by mechanisms related, at least to some extent, to DNA methylation.

Our recent porcine endometrial transcriptome analysis showed a significant effect of E_2_ [4]. The secretion profile of prostaglandin E_2_ (PGE_2_) by the porcine endometrium and conceptuses is similar to the estrogen secretion by porcine conceptuses [23–25]. We recently found that PGE_2_ is an important factor that augments the effect of E_2_ in porcine endometrium and induces changes within the endometrial transcriptome similar to those observed for the effect of embryos during pregnancy establishment [23–25]. Functional annotation clustering analyses linked these alterations to the processes important for pregnancy establishment (i.e., tissue remodeling, regulation of immune response, cell proliferation, differentiation, and migration). Interestingly, within the identified processes, we found those that are related to DNA methylation [4, 25]. Moreover, studies on the epigenetic effect of exogenous estradiol administration revealed E_2_-induced subtle but consistent hypomethylation of DNA within the promoter region of cyclin-dependent kinase inhibitor 2D (*CDKN2D*) and in exon 1 of phosphoserine aminotransferase 1 (*PSAT1*) not only in the porcine endometrium and corpora lutea (CL) but also in F1 preimplantation embryos at day 10 of development [26]. Overall, the knowledge of epigenetic processes regulating the physiological function of the endometrium and understanding the role of embryonic signals in this regulation is limited in any species. Therefore, two questions arise, namely (1) if the gene expression changes in the porcine endometrium are related to differential DNA methylation and (2) if E_2_ of conceptus origin can induce changes in DNA methylation levels of differentially expressed genes. Furthermore, since PGE_2_ augments the effect of E_2_ in changes of the endometrial transcriptome, PGE_2_ may also support the epigenetic effects of E_2_. Therefore, the aims of the present study were (1) to determine the effect of E_2_ alone and E_2_ acting simultaneously with PGE_2_ on endometrial mRNA and protein expression of DNMT1, DNMT3A, and DNMT3B and to compare these effects to those occurring in the porcine endometrium on day 12 of pregnancy opposed to day 12 of the estrous cycle; (2) to assess the effect of E_2_ and PGE_2_ on the activity of DNMTs in the porcine endometrium; and (3) to evaluate whether E_2_ acting alone or simultaneously with PGE_2_ induced changes in DNA methylation level of selected pregnancy-related genes in the endometrium.

## Materials and methods

All procedures involving animals in experiment 1 and in experiment 2 were conducted in accordance with the national guidelines for agricultural animal care and were approved by the Animal Ethics Committee, University of Warmia and Mazury in Olsztyn, Poland, permission No. 17/2008.

### Experiment 1: in vivo model of conceptus signaling

The effects of E_2_, PGE_2_, and an E_2_ + PGE_2_ treatment were studied using a previously described in vivo model [4, 25, 27]. Prepubertal crossbred gilts of 6 months of age and a similar genetic background (Pietrain × Duroc) after the second natural estrus following puberty were treated hormonally with 750 IU of Pregnant Mare Serum Gonadotrophin (PMSG; Folligon, Intervet, Boxmeer, The Netherlands) and 500 IU of human chorionic gonadotropin (hCG; Chorulon, Intervet) given 72 h later to induce estrus. Subsequently, between days 12 and 14 of the estrous cycle, 10 mg of prostaglandin F2α (PGF2α; Dinolytic; Pfizer, Warsaw, Poland) was injected intramuscularly to induce estrus. After 16 h, 10 mg of PGF2α was injected simultaneously with 750 IU of PMSG. After 72 h, 500 IU of hCG was given intramuscularly. On days 8–9 of the third estrous cycle following puberty, animals were subjected to a surgical procedure in which uterine horns were exposed and a cannula was introduced directly into the uterine lumen of each horn at a distance of 10–15 cm from the isthmus. In order to imitate hormone delivery by conceptuses, the cannula was perforated along its length as reported previously [28] with some modifications. After recovery of the surgery, animals were divided into following groups: (1) control (*n* = 7)—received a placebo (5 mL of 0.1% v/v ethanol saline) infusions into both uterine horns; (2) E_2__833 ng (*n* = 5)—received 833 ng of E_2_ per infusion into one uterine horn and a placebo infusion into contralateral uterine horn; (3) E_2__33.3 μg (*n* = 5)—received 33.3 μg of E_2_ per infusion into one uterine horn and placebo infusion into contralateral uterine horn; (4) PGE_2_ (*n* = 6)—received 200 μg of PGE_2_ per infusion into one uterine horn and a placebo infusion into contralateral uterine horn; and (5) E_2_ + PGE_2_ (*n* = 6)—received 33.3 μg of E_2_ simultaneously with 200 μg of PGE_2_ per infusion into one uterine horn and placebo infusion into contralateral uterine horn. Doses of hormones used were similar to those previously published [28–30]. In our previous report [4], we evidenced that a lower dose of E_2_ (833 ng/infusion) resulted in alterations within the endometrial transcriptome similar to pregnancy effect. However, a higher dose of E_2_ (33.3 μg/infusion) induced greater changes in the global gene expression profile that were more similar to changes identified in the endometrial transcriptome on day 12 of the pregnancy. Hormone or placebo infusions were administered every 4 h for 24 h on days 11–12 after the onset of estrus. After the experiment, animals were slaughtered, and endometrial samples were collected and snap-frozen in liquid nitrogen. Inflammatory changes and/or fluid accumulation excluded collected uteri from further analyses.

### Experiment 2: animal model ex vivo

Gilts on day 12 of the estrous cycle and pregnancy were used as reference groups. For this, prepubertal crossbred gilts with similar age (~6 months) and genetic background (Pietrain × Duroc) were observed for the onset of the estrous cycle. After two natural estrous cycles, gilts were divided into two groups: cyclic and pregnant. Gilts assigned to the “pregnant” group were mated with a boar twice at 24 h and 48 h after the onset of estrus. On day 12 of the estrous cycle (*n* = 7) and pregnancy (*n* = 7), gilts were slaughtered in the local abattoir. Uterine horns collected from pregnant gilts were flushed with sterile phosphate-buffered saline to collect the embryos. Pregnancy was confirmed by the presence of conceptuses. Endometrial tissue was dissected from the myometrium by scissors and snap-frozen in liquid nitrogen.

### The effects of E2 and PGE2 on endometrial DNMT gene and protein expression

#### Quantitative real-time RT-PCR

The endometrial *DNMT1*, *DNMT3A*, and *DNMT3B* gene expression were determined using quantitative PCR (qPCR, real-time RT-PCR), as described previously [31]. Detailed information of qPCR procedure has been provided in Supplementary Information.

#### Western blot

The effects of E_2_, PGE_2_, and E_2_ acting simultaneously with PGE_2_ on endometrial DNMT1, DNMT3A, and DNMT3B protein expression were studied by using Western blot as described earlier [25]. Equal amounts of nuclear protein extracts (40 μg) were dissolved in SDS gel-loading buffer (50 mM/L Tris–HCl, pH 6.8; 4% SDS, 20% glycerol, and 2% β-mercaptoethanol), then denatured at 95°C for 4 min, and separated on 4–20% stain-free polyacrylamide gels (Bio-Rad, Hercules, CA, USA). Separated proteins were electroblotted onto 0.2 mm PVDF membrane (Millipore, Burlington, MA, USA) in transfer buffer (20 mM/L Tris–HCl buffer, pH 8.2; 150 mM/L glycine, and 20% methanol). Membranes were then blocked in 5% non-fat dry milk in Tris-buffered saline buffer (TBS-T, containing 0.1% Tween-20) for 1.5 h at room temperature. After blocking, the membranes were incubated overnight with primary antibodies against DNMT1, DNMT3A and DNMT3B or negative isotype IgG controls (Supplementary Table 2). Following incubation, membranes were washed three times in TBST-T and incubated with anti-rabbit or anti-mouse secondary antibodies (Bio-Rad; Supplementary Table 2) dissolved 1:20 000 in TBS-T, in room temperature for 90 min. After incubation, membranes were also washed three times in TBS-T. Immune complexes were visualized using chemiluminescent HRP visualization procedure. Blots were photographed using ChemiDoc Imaging System (Bio-Rad) and archived in graphical format files. Blots were analyzed using ImageLab (6.0) software (Bio-Rad). Signal intensities reflecting target protein expression were normalized against total protein content [32] detected on the entire blot using stain-free technology and Image Lab 6.0 (Bio-Rad) algorithm [33].

### The effect of E2 and PGE_2_ on endometrial DNMT activity

Using the in vivo (experiment 1) and the ex vivo models (experiment 2), we studied the effect of E_2_ acting alone and simultaneously with PGE_2_ and the effect of pregnancy on DNMTs enzyme activity. Nuclear protein fractions were extracted from endometrial samples using the Nuclear Extraction Kit (Cayman Chemical, Ann Arbor, MI, USA) according to the manufacturer’s protocol. The protein concentration in homogenates was determined using the Bradford assay [34]. The DNMT activity in analyzed samples was determined by commercially available DNMT Activity/Inhibition Assay (Active Motif, Carlsbad, CA, USA) according to the manufacturer’s instructions. Each measurement was performed in duplicates. The DNMT activity (OD/h/mg) was calculated as follows: (average sample OD – average blank OD)/protein amount (10 μg) × time (2 h).

### The effect of E_2_ acting alone and simultaneously with PGE_2_ on local DNA methylation in porcine endometrium in vivo

#### DNA/RNA isolation and bisulfite conversion

Total DNA and RNA from collected endometrial tissues were extracted with the AllPrep DNA/RNA Mini Kit (Qiagen, Hilden, Germany) following the manufacturer’s instructions. After extraction, DNA was quantified using the NanoDrop 1000 and the quality was assessed spectrophotometrically and by electrophoresis. In order to convert unmethylated cytosine residuals to uracil, isolated DNA was converted by using EpiTect Bisulfite Kit (Qiagen, Hilden, Germany) according to the manufacturer’s protocol. Bisulfite-converted DNA (bc-DNA) was then quantified using NanoDrop 1000 spectrophotometer.

#### DNA methylation assays

Genes important for processes accompanying development of early pregnancy such as metabolism (*ADH1C*), cell cycle regulation (*PSAT1*), cell proliferation (*RASFF1* and *DMBT1*), and tissue remodeling (*ADAMTS20*, *BGN* and *WNT5A*) [4, 25, 26, 35] have been selected for local methylation analyses. DNA methylation assays for selected sequences of *ADH1C*, *BGN*, *PSAT1*, and *RSSF1* genes were designed earlier [26, 35]. The region of interest for the DNA methylation assays of *ADAMTS20*, *DMBT1*, and *WNT5A* was chosen based on results from methylome sequencing of porcine corpora lutea [36] and embryos [37]. Briefly, genes of interest were selected based on our results from endometrial transcriptome profiling [4, 25] and compared with genes annotated to DMRs identified in porcine CLs and/or embryos [36, 37]. DNA sequences (promoter regions and gene bodies) of target genes were browsed to identify fragments rich in CpG sites. Regions for analyses were selected based on the consensus between the highest possible number of CpG sites within analyzed sequences and the possibility of picking the primers allowing for amplification of specific PCR product. Detailed information on the studied DNA fragments and genomic localization of analyzed CpG sites are presented in the Supplementary Figure 1.

#### Bisulfite pyrosequencing

The bisulfite pyrosequencing was performed as described previously [38]. In total, 48 samples were bisulfite converted. Control group: number of animals *n* = 4, number of samples = 8 (4 left horn and 4 right horn); E_2__833 ng group: number of animals *n* = 5, number of samples = 10 (5 hormone-treated horn, 5 placebo-treated horn); E_2__33.3 μg group: number of animals *n* = 4, number of samples = 8 (4 hormone-treated horn, 4 placebo-treated horn); E_2_ + PGE_2_ group: number of animals *n* = 6, number of samples = 12 (6 hormone-treated horn, 6 placebo-treated horn); day 12 of the estrous cycle: number of animals *n* = 5, number of samples = 5; day 12 of pregnancy: number of animals *n* = 5, number of samples = 5. The primers for the selected DNA fragment amplification and PyroMark assays were designed using the PyroMark Assay design Software 2.0 (Qiagen, Hilden, Germany; Supplementary Table 3). The annealing temperatures were as follows: 55°C, 57.5°C, 57°C, 51°C, 58°C, 56°C, and 57°C for the *ADAMTS20*, *ADH1C*, *BGN*, *DMBT1*, *PSAT1*, *RASSF1*, and *WNT5A* assays, respectively. The specificity of amplified product was validated by gel electrophoresis. The levels of CpG (*ADAMTS20*, *ADH1C*, *BGN*, *DMBT1*) or CpN (*PSAT1*, *RASSF1*) methylation were quantified using the PyroMark Q48 Autoprep System (Qiagen) and the PyroMark Q48 Advanced CpG Reagents (Qiagen). Methylation values [%] were calculated with the PyroMark Q48 Autoprep 2.4.1 Software (Qiagen).

### The effects of E_2_ and PGE_2_ on endometrial gene expression

#### Real-time RT-PCR

The gene expression of ADAM metallopeptidase with thrombospondin type 1 motif 20 (*ADAMTS20*), deleted in malignant brain tumors 1 (*DMBT1*) and Wnt family member 5A (*WNT5A*) in porcine endometrium in response to hormone treatment in vivo (experiment 1) and in endometrial samples collected on day 12 of pregnancy and the estrous cycle (experiment 2), was determined in previous studies [4, 25]. In the present study, results were reanalyzed including PGE_2_-treated group. Endometrial expression of Alcohol Dehydrogenase 1C (*ADH1C*), Biglycan (*BGN*), Phosphoserine Aminotransferase 1 (*PSAT1*), and Ras Association Domain Family Member 1 (*RASSF1*) genes towards hormonal treatment and on day 12 of pregnancy and the estrous cycle was determined using qPCR as described earlier [4, 25]. Detailed information of qPCR procedure has been provided in Supplementary Information.

### Statistical analyses

Results from endometrial gene and protein expression studies, DNMT activity, and from DNA methylation studies in samples collected from gilts after experiment 1 were analyzed using two-way ANOVA, followed by Tukey post-test. Main effects: the effect of treatment between groups: control versus hormone-treated and the effect of the site of hormone administration (i.e., placebo-treated uterine horn vs. hormone-treated uterine horn) on endometrial gene and protein expression and the levels of DNA methylation were assessed. A main effect of treatment was detected, whereas there was no main effect of the site of hormone administration (except for mRNA expression of *ADAMTS20*). Therefore, we analyzed the main effect of treatment by Tukey post-test (endometria of control gilts vs endometria of E_2_-, PGE_2_-, and E_2_ + PGE_2_-treated gilts).

Gene and protein expression, DNMT activity as well as methylation levels of selected DNA sequences in endometrial samples collected from gilts on day 12 of the estrous cycle and pregnancy (experiment 2) were analyzed using *T*-tests.

The correlation analyses were performed using Pearson correlation. All data were tested for normality and homoscedasticity. Log-transformation of data was applied before performing parametric tests when needed. Differences were considered as statistically significant at the 95% confidence level (*P* < 0.05). All statistical analyses were conducted using GraphPad PRISM v. 9.0. software (GraphPad Software Inc., San Diego, CA, USA).

## Results

### DNMT gene and protein expression in the porcine endometrium in vivo is regulated by E_2_ and PGE_2_ and by the presence of conceptuses

**Figure 1:**
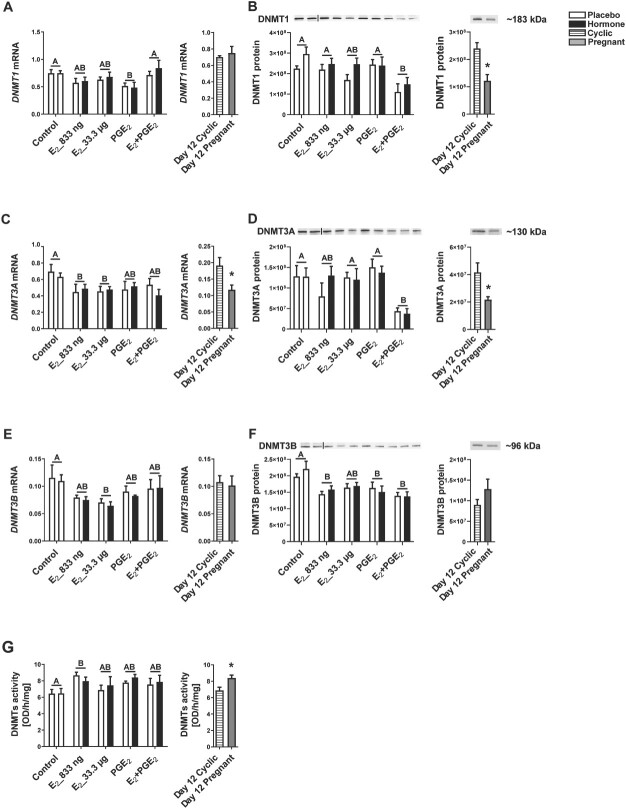
Endometrial mRNA and protein expression of DNMT 1, 3A, and 3B: DNMT1 (A, B), DNMT3A (C, D), and DNMT3B (E, F), and endometrial DNMT activity (G) in response to estradiol-17β (E_2_), prostaglandin E_2_ (PGE_2_), and E_2_ + PGE_2_ treatment in vivo and in gilts on day 12 of the estrous cycle and pregnancy. Control: Gilts received a placebo infusion into both uterine horns. E_2__833 ng: gilts received infusions of a placebo into one randomly selected horn and E_2_ (833 ng/infusion) into the contralateral horn. E_2__33.3 μg: Gilts received infusions of a placebo into one randomly selected uterine horn and E_2_ (33.3 μg/infusion) into the contralateral horn. PGE_2_: Gilts received infusions of a placebo into one randomly selected horn and PGE_2_ (200 μg/infusion) into the contralateral horn. E_2_ + PGE_2_: Gilts received infusions of either placebo into one randomly selected horn or E_2_ (33.3 μg/infusion) together with PGE_2_ (200 μg/infusion) into the contralateral horn. As a reference, gilts on day 12 of the estrous cycle (Day 12 Cyclic) and pregnancy (Day 12 Pregnant) were included. Data are expressed as the mean ± SEM. While the main effect of treatment was detected, there was no main effect of the site of hormone administration. Different letters indicate statistically significant differences between the control- and the hormone-treated groups (*P* < 0.05). The asterisk indicates statistical differences between gilts on day 12 of the estrous cycle and pregnancy (*P* < 0.05).

The effects of E_2_, PGE_2_, and E_2_ + PGE_2_ and the effect of reproductive status (pregnant vs. cyclic) on endometrial mRNA and protein expression of DNA methyltransferases 1, 3A, and 3B were analyzed. We found an effect of treatment (hormone-treated group vs. control-treated group) for the mRNA expression of *DNMT1*, *DNMT3A*, and *DNMT3B* (*P* < 0.01 for *DNMT1*; *P* < 0.05 for *DNMT3A* and *DNMT3B*; Figure 1) and for protein expression of DNMT1, DNMT3A, and DNMT3B (*P* < 0.001 for DNMT1 and DNMT3B; *P* < 0.0005 for DNMT3A; Figure 1B, D, and F, respectively). There was no main effect of the site of hormone administration (hormone-treated uterine horn vs. placebo-treated uterine horn) on mRNA and protein expression of DNMTs.

PGE_2_ administered alone into the uterine lumen significantly decreased the endometrial expression of the *DNMT1* gene (*P* < 0.05; Figure 1A). E_2__833 ng administered alone significantly lowered endometrial expression of the *DNMT3A* gene (*P* < 0.05; Figure 1C). Intrauterine infusion of E_2__33.3 μg significantly decreased the expression of the *DNMT3A* and *DNMT3B* (*P* < 0.05) genes (Figure 1C and E). In porcine endometrial samples collected on day 12 of pregnancy, the expression of the *DNMT3A* gene was significantly lower (*P* < 0.05) than in endometrial samples collected on day 12 of the estrous cycle (Figure 1C).

Both PGE_2_ and E_2__833 ng administered alone into the uterine lumen had no effect on the endometrial expression of DNMT1 and DNMT3A proteins (Figure 1B and D; Supplementary Figure 2A and B). However, PGE_2_ administered simultaneously with E_2_ significantly decreased the endometrial levels of DNMT1 and DNMT3A proteins (*P* < 0.05; Figure 1B and D). Both PGE_2_ and E_2__833 ng administered alone and E_2_ + PGE_2_ significantly decreased the endometrial protein expression of DNMT3B (*P* < 0.05; Fig. 1F, Supplementary Fig. 2C). Expression of the DNMT1 and DNMT3A proteins was significantly lower in endometrial samples collected from the gilts on day 12 of pregnancy compared to endometrial samples collected from the gilts on day 12 of the estrous cycle (*P* < 0.05; Figure 1B and D).

### E_2_ and the presence of conceptuses stimulate endometrial DNMT activity in vivo

The expression of DNMT mRNA and protein was affected by E_2_ and PGE_2_ treatment as well as by pregnancy; therefore, we determined the effect of E_2_ and PGE_2_ and the effect of pregnancy on endometrial DNMT activity. We found the effect of treatment (*P* < 0.05) and the effect of reproductive status (*P* < 0.05; Figure 1G). The activity of DNMT enzymes in the porcine endometrium was significantly higher (*P* < 0.05) in samples collected from E_2__833 ng-treated gilts (Figure 1G). Additionally, we found increased activity of DNMT in endometrial samples collected from the gilts on day 12 of pregnancy compared to endometrial samples collected from the gilts on day 12 of the estrous cycle (*P* < 0.05; Figure 1G).

### The effect of E_2_ and/or PGE_2_ and the effect of pregnancy on DNA methylation patterns of selected gene sequences in the porcine endometrium

**Figure 2:**
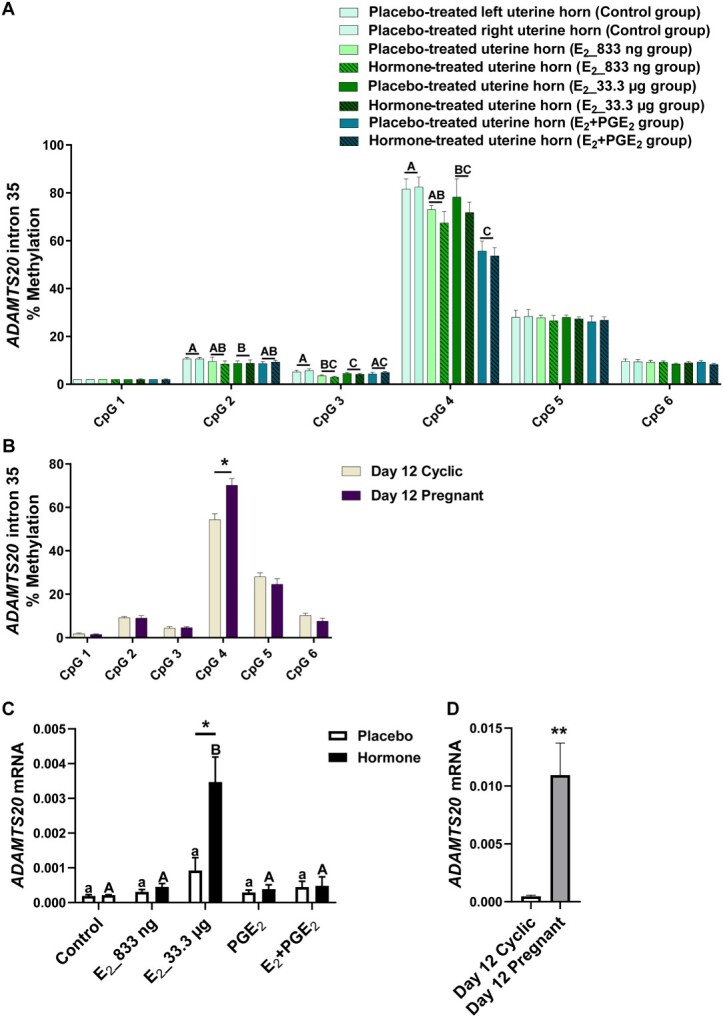
Methylation levels of single CpG sites localized within *ADAMTS20* intron 35 in response to estradiol-17β (E_2_) administered alone (833 ng or 33.3 μg/infusion) or simultaneously with prostaglandin E_2_ (PGE_2_, 200 μg/infusion) in vivo (A) and in gilts on day 12 of the estrous cycle and pregnancy (B). Endometrial mRNA abundance of *ADAMTS20* in response to E_2_, PGE_2_, and E_2_ + PGE_2_ treatment in vivo (C) and in gilts on day 12 of the estrous cycle and pregnancy (D). Control: Gilts received a placebo infusion into both uterine horns. E_2__833 ng: Gilts received infusions of a placebo into one randomly selected horn and E_2_ (833 ng/infusion) into the contralateral horn. E_2__33.3 μg: Gilts received infusions of a placebo into one randomly selected uterine horn and E_2_ (33.3 μg/infusion) into the contralateral horn. PGE_2_: Gilts received infusions of a placebo into one randomly selected horn and PGE_2_ (200 μg/infusion) into the contralateral horn. E_2_ + PGE_2_ gilts received infusions of either placebo into one randomly selected horn or E_2_ (33.3 μg/infusion) together with PGE_2_ (200 μg/infusion) into the contralateral horn. As a reference, gilts on day 12 of the estrous cycle (Day 12 Cyclic) and pregnancy (Day 12 Pregnant) were included. Data are expressed as the mean ± SEM. Different letters in panel A indicate statistically significant differences between the control- and the hormone-treated groups (*P* < 0.05). Different capital letters in panel C indicate statistically significant differences between hormone-treated uterine horns of the hormone-treated groups and placebo-treated horns of the control group (*P* < 0.05). Asterisks in panel C indicate statistically significant differences between placebo- and hormone-treated horns within the same group (Hormone-treated groups). Asterisks in panels B and D indicate significant differences (*P* < 0.05) between gilts on day 12 of the estrous cycle and pregnancy. Results of mRNA expression on day 12 of the estrous cycle and pregnancy presented on panel D have been published in [4].

**Figure 3:**
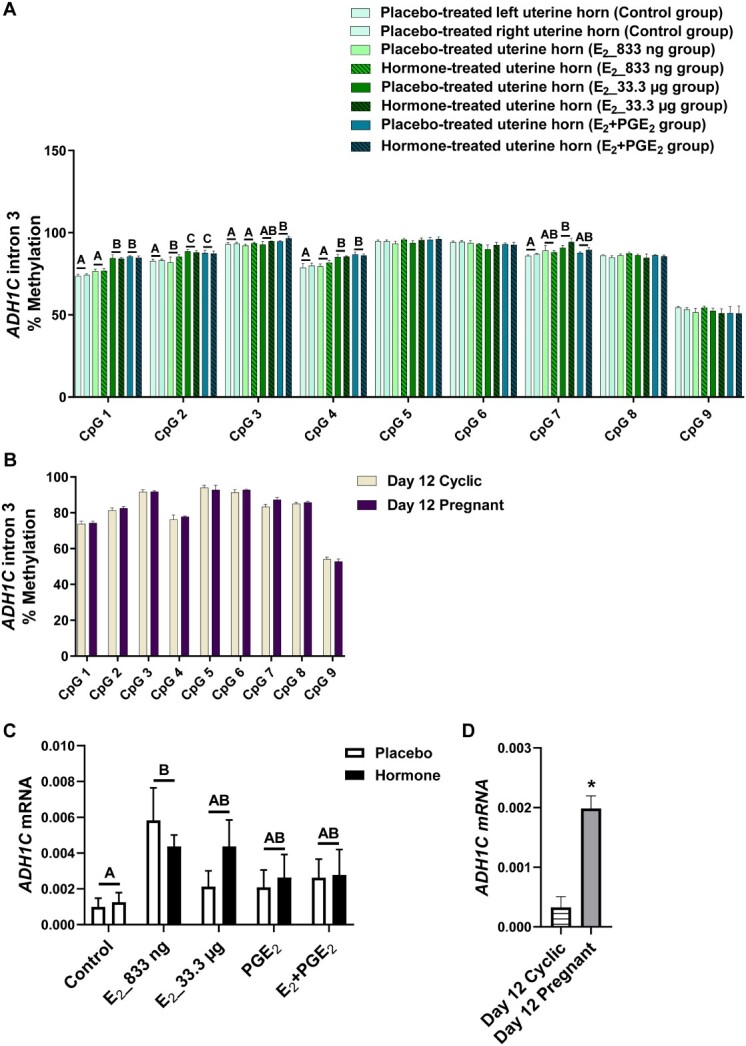
Methylation levels of single CpG sites localized within *ADH1C* intron 3 in response to estradiol-17β (E_2_) administered alone (833 ng or 33.3 μg/infusion) or simultaneously with prostaglandin E_2_ (PGE_2_, 200 μg/infusion) in vivo (A) and in gilts on day 12 of the estrous cycle and pregnancy (B). Endometrial mRNA expression of *ADH1C* in response to E_2_, prostaglandin PGE_2_, and E_2_ + PGE_2_ treatment in vivo (C) and in gilts on day 12 of the estrous cycle and pregnancy (D). Control: Gilts received a placebo infusion into both uterine horns. E_2__833 ng: Gilts received infusions of a placebo into one randomly selected horn and E_2_ (833 ng/infusion) into the contralateral horn. E_2__33.3 μg: Gilts received infusions of a placebo into one randomly selected uterine horn and E_2_ (33.3 μg/infusion) into the contralateral horn. PGE_2_: Gilts received infusions of a placebo into one randomly selected horn and PGE_2_ (200 μg/infusion) into the contralateral horn. E_2_ + PGE_2_: Gilts received infusions of either placebo into one randomly selected horn or E_2_ (33.3 μg/infusion) together with PGE_2_ (200 μg/infusion) into the contralateral horn. As reference, gilts on day 12 of the estrous cycle (Day 12 Cyclic) and pregnancy (Day 12 Pregnant) were included. Data are expressed as the mean ± SEM. While the main effect of treatment was detected, there was no main effect of the site of hormone administration. Different letters indicate statistically significant differences between the control- and the hormone-treated groups (*P* < 0.05). The asterisk in panel D indicates statistical differences between gilts on day 12 of the estrous cycle and pregnancy (*P* < 0.05) [4].

**Figure 4:**
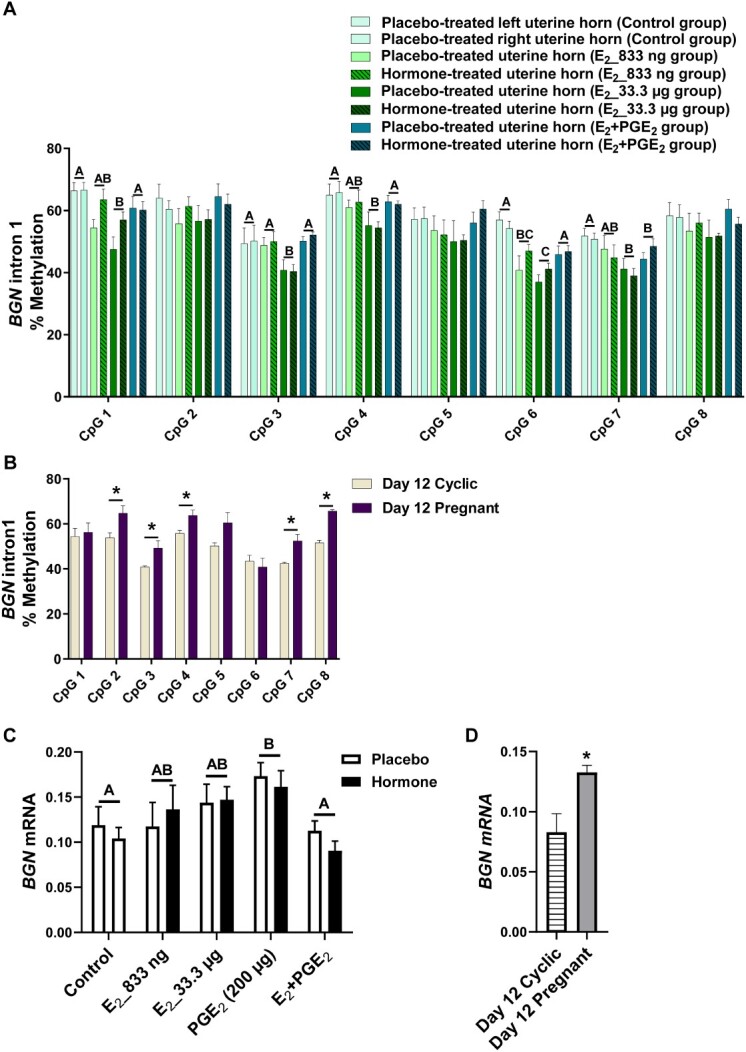
Methylation levels of single CpG sites localized within *BGN* intron 1 in response to estradiol-17β (E_2_) administered alone (833 ng or 33.3 μg/infusion) or simultaneously with prostaglandin E_2_ (PGE_2_, 200 μg/infusion) in vivo (A) and in gilts on day 12 of the estrous cycle and pregnancy (B). Endometrial mRNA expression of *BGN* in response to E_2_, PGE_2_, and E_2_ + PGE_2_ treatment in vivo (C) and in gilts on day 12 of the estrous cycle and pregnancy (D). Control: Gilts received a placebo infusion into both uterine horns. E_2__833 ng: Gilts received infusions of a placebo into one randomly selected horn and E_2_ (833 ng/infusion) into the contralateral horn. E_2__33.3 μg: Gilts received infusions of a placebo into one randomly selected uterine horn and E_2_ (33.3 μg/infusion) into the contralateral horn. PGE_2_: Gilts received infusions of a placebo into one randomly selected horn and PGE_2_ (200 μg/infusion) into the contralateral horn. E_2_ + PGE_2_: Gilts received infusions of either placebo into one randomly selected horn or E_2_ (33.3 μg/infusion) together with PGE_2_ (200 μg/infusion) into the contralateral horn. As reference, gilts on day 12 of the estrous cycle (Day 12 Cyclic) and pregnancy (Day 12 Pregnant) were included. Data are expressed as the mean ± SEM. While the main effect of treatment was detected, there was no main effect of the site of hormone administration. Different letters indicate statistically significant differences between the control- and the hormone-treated groups (*P* < 0.05). Asterisks in panels B and D indicate statistical differences between gilts on day 12 of the estrous cycle and pregnancy (*P* < 0.05) [4].

**Figure 5:**
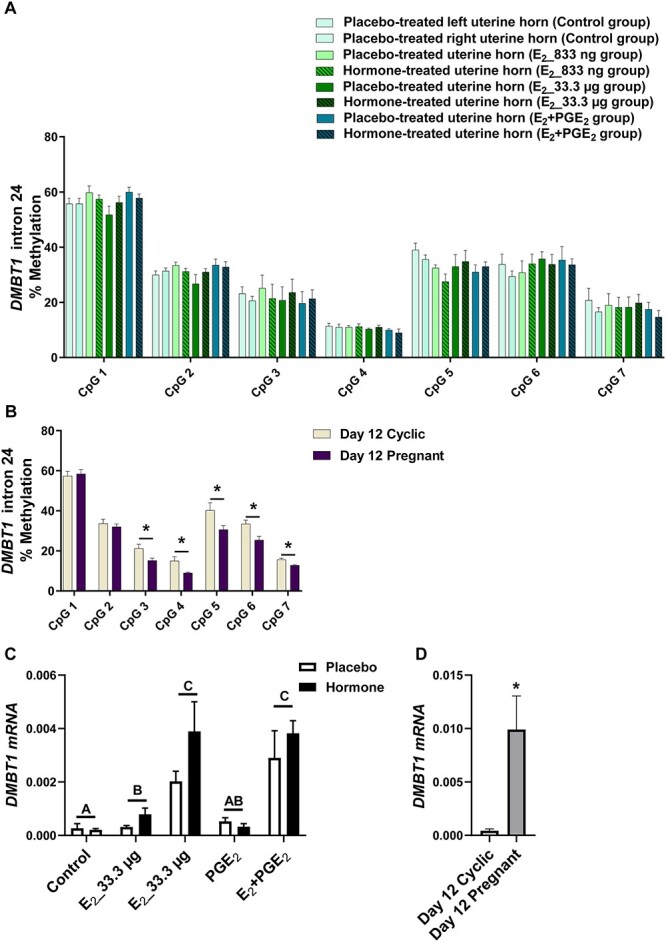
Methylation levels of single CpG sites localized within *DMBT1* intron 24 in response to estradiol-17β (E_2_) administered alone (833 ng or 33.3 μg/infusion) or simultaneously with prostaglandin E_2_ (PGE_2_, 200 μg/infusion) in vivo (A) and in gilts on day 12 of the estrous cycle and pregnancy (B). Endometrial mRNA expression of *DMBT1* in response to E_2_, PGE_2_, and E_2_ + PGE_2_ treatment in vivo (C) and in gilts on day 12 of the estrous cycle and pregnancy (D). Control: Gilts received a placebo infusion into both uterine horns. E_2__833 ng: Gilts received infusions of a placebo into one randomly selected horn and E_2_ (833 ng/infusion) into the contralateral horn. E_2__33.3 μg: Gilts received infusions of a placebo into one randomly selected uterine horn and E_2_ (33.3 μg/infusion) into the contralateral horn. PGE_2_: Gilts received infusions of a placebo into one randomly selected horn and PGE_2_ (200 μg/infusion) into the contralateral horn. E_2_ + PGE_2_: Gilts received infusions of either placebo into one randomly selected horn or E_2_ (33.3 μg/infusion) together with PGE_2_ (200 μg/infusion) into the contralateral horn. As reference, gilts on day 12 of the estrous cycle (Day 12 Cyclic) and pregnancy (Day 12 Pregnant) were included. Data are expressed as the mean ± SEM. While the main effect of treatment was detected affecting the mRNA level, there was no main effect of the site of hormone administration. Different letters indicate statistically significant differences between the control- and the hormone-treated groups (*P* < 0.05). Asterisks in panels B and D indicate statistical differences between gilts on day 12 of the estrous cycle and pregnancy (*P* < 0.05). Results of mRNA expression on day 12 of the estrous cycle and pregnancy presented in panel D have been published in [4].

**Figure 6:**
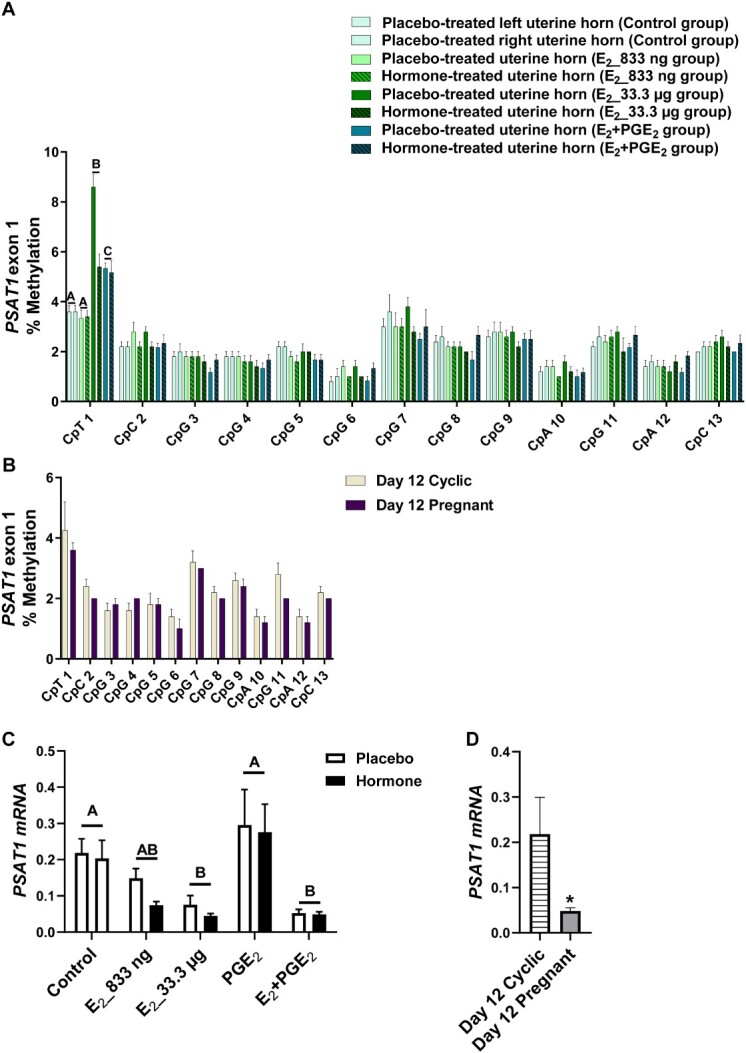
Methylation levels of single CpN sites localized within *PSAT1* exon 1 in response to estradiol-17β (E_2_) administered alone (833 ng or 33.3 μg/infusion) or simultaneously with prostaglandin E_2_ (PGE_2_, 200 μg/infusion) in vivo (A) and in gilts on day 12 of the estrous cycle and pregnancy (B). Endometrial mRNA expression of *PSAT1* in response to E_2_, PGE_2_, and E_2_ + PGE_2_ treatment in vivo (C) and in gilts on day 12 of the estrous cycle and pregnancy (D). Control: Gilts received a placebo infusion into both uterine horns. E_2__833 ng: Gilts received infusions of a placebo into one randomly selected horn and E_2_ (833 ng/infusion) into the contralateral horn. E_2__33.3 μg: Gilts received infusions of a placebo into one randomly selected uterine horn and E_2_ (33.3 μg/infusion) into the contralateral horn. PGE_2_: Gilts received infusions of a placebo into one randomly selected horn and PGE_2_ (200 μg/infusion) into the contralateral horn. E_2_ + PGE_2_: Gilts received infusions of either placebo into one randomly selected horn or E_2_ (33.3 μg/infusion) together with PGE_2_ (200 μg/infusion) into the contralateral horn. As reference, gilts on day 12 of the estrous cycle (Day 12 Cyclic) and pregnancy (Day 12 Pregnant) were included. Data are expressed as the mean ± SEM. While the main effect of treatment was detected, there was no main effect of the site of hormone administration. Different letters indicate statistically significant differences between the control- and the hormone-treated groups (*P* < 0.05). The asterisk in panel D indicates statistical differences between gilts on day 12 of the estrous cycle and pregnancy (*P* < 0.05).

**Figure 7:**
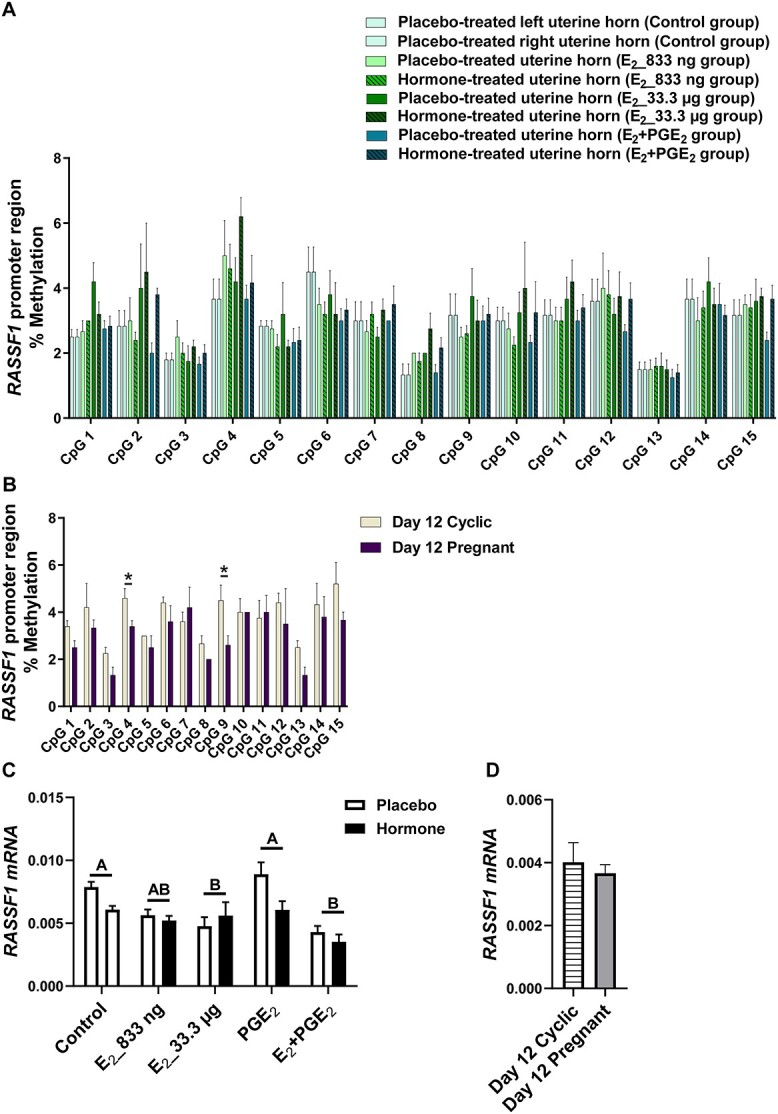
Methylation levels of single CpG sites localized within *RASSF1* promoter region in response to estradiol-17β (E_2_) administered alone (833 ng or 33.3 μg/infusion) or simultaneously with prostaglandin E_2_ (PGE_2_, 200 μg/infusion) in vivo (A) and in gilts on day 12 of the estrous cycle and pregnancy (B). Endometrial mRNA expression of *RASSF1* in response to E_2_, PGE_2_, and E_2_ + PGE_2_ treatment in vivo (C) and in gilts on day 12 of the estrous cycle and pregnancy (D). Control: Gilts received a placebo infusion into both uterine horns. E_2__833 ng: Gilts received infusions of a placebo into one randomly selected horn and E_2_ (833 ng/infusion) into the contralateral horn. E_2__33.3 μg: Gilts receiving infusions of a placebo into one randomly selected uterine horn and E_2_ (33.3 μg/infusion) into the contralateral horn. PGE_2_: Gilts receiving infusions of a placebo into one randomly selected horn and PGE_2_ (200 μg/infusion) into the contralateral horn. E_2_ + PGE_2_: Gilts receiving infusions of either placebo into one randomly selected horn or E_2_ (33.3 μg/infusion) together with PGE_2_ (200 μg/infusion) into the contralateral horn. As reference, gilts on day 12 of the estrous cycle (Day 12 Cyclic) and pregnancy (Day 12 Pregnant) were included. Data are expressed as the mean ± SEM. While the main effect of treatment was detected on mRNA level, there was no main effect of the site of hormone administration. Different letters indicate statistically significant differences between the control- and the hormone-treated groups (*P* < 0.05). Asterisks in panel B indicate statistical differences between gilts on day 12 of the estrous cycle and pregnancy (*P* < 0.05).

**Figure 8:**
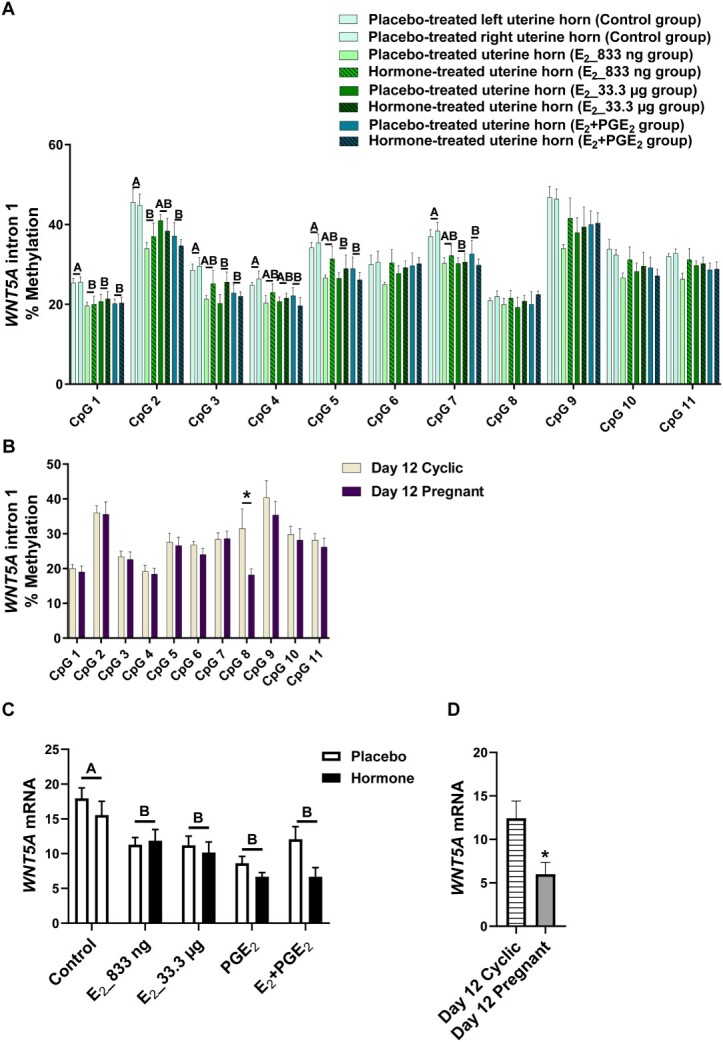
Methylation levels of single CpG sites localized within *WNT5A* intron 1 in response to estradiol-17β (E_2_) administered alone (833 ng or 33.3 μg/infusion) or simultaneously with prostaglandin E_2_ (PGE_2_, 200 μg/infusion) in vivo (A) and in gilts on day 12 of the estrous cycle and pregnancy (B). Endometrial mRNA expression of *WNT5A* in response to E_2_, PGE_2_, and E_2_ + PGE_2_ treatment in vivo (C) and in gilts on day 12 of the estrous cycle and pregnancy (D). Control: Gilts received a placebo infusion into both uterine horns. E_2__833 ng: Gilts received infusions of a placebo into one randomly selected horn and E_2_ (833 ng/infusion) into the contralateral horn. E_2__33.3 μg: Gilts receiving infusions of a placebo into one randomly selected uterine horn and E_2_ (33.3 μg/infusion) into the contralateral horn. PGE_2_: Gilts receiving infusions of a placebo into one randomly selected horn and PGE_2_ (200 μg/infusion) into the contralateral horn. E_2_ + PGE_2_: Gilts receiving infusions of either placebo into one randomly selected horn or E_2_ (33.3 μg/infusion) together with PGE_2_ (200 μg/infusion) into the contralateral horn. As reference, gilts on day 12 of the estrous cycle (Day 12 Cyclic) and pregnancy (Day 12 Pregnant) were included. Data are expressed as the mean ± SEM. While the main effect of treatment was detected, there was no main effect of the site of hormone administration. Different letters indicate statistically significant differences between the control- and the hormone-treated groups (*P* < 0.05). Asterisks in panels B and D indicate statistical differences between gilts on day 12 of the estrous cycle and pregnancy (*P* < 0.05). Results of mRNA expression on day 12 of the estrous cycle and pregnancy presented in panel D have been published in [4].

Given the differences in DNMT activity during pregnancy or infusion of E_2_ and PGE_2_, both alone and in combination, we investigated the DNA methylation of target genes. We studied whether E_2_ alone and E_2_ acting simultaneously with PGE_2_ affected the methylation levels of CpGs of selected gene sequences. We detected the effect of treatment (*P* < 0.05) for CpGs in *ADAMTS20* (Figure 2A), *ADH1C* (Figure 3A), *BGN* (Figure 4A), *PSAT1* (Figure 6A), and *WNT5A* (Figure 8A). There was no main effect of the site of hormone administration (hormone-treated uterine horn vs. placebo-uterine horn) on DNA methylation. We also found the effect of the reproduction status (pregnant vs. cyclic; *P* < 0.05) on methylation levels of CpG in *ADAMTS20* (Figure 2A), *BGN* (Figure 4A), *DMBT1* (Figure 5A), *RASSF1* (Figure 7A), and in *WNT5A* (Figure 8A).

E_2__833 ng administered alone resulted in reduced methylation levels of CpG at position 3 in the *ADAMTS20* intron 35 sequence (*P* < 0.05; Figure 2A); CpG at position 6 in the *BGN* intron 1 sequence (*P* < 0.05; Figure 4A); CpGs at positions 1 and 2 in the *WNT5A* intron 1 sequence (*P* < 0.05; Figure 8A); and increased methylation of CpGs at position 2 in the *ADH1C* intron 3 sequence (*P* < 0.05; Figure 3A). Administration of E_2__33 μg alone lowered methylation levels of CpGs at positions 2, 3, and 4 of the *ADAMTS20* intron 35 sequence (*P* < 0.05; Figure 2A); CpGs at positions 1, 3, 4, 6, and 7 of the *BGN* intron 1 sequence (*P* < 0.05; Figure 4A); and CpGs at positions 1, 3, 5, and 7 of the *WNT5A* intron 1 sequence (*P* < 0.05; Figure 8A) and induced hypermethylation of CpGs at positions 1, 2, 4, and 7 of the *ADH1C* intron 3 sequence (*P* < 0.05; Figure 3A) and CpT at position 1 of the *PSAT1* exon 1 sequence (*P* < 0.05; Figure 6A). E_2_ administered simultaneously with PGE_2_ led to hypomethylation of CpG at position 4 in the *ADAMTS20* intron 35 sequence (Figure 2A); CpG at position 7 in the *BGN* intron 1 sequence (*P* < 0.05; Figure 4A); and CpGs at positions 1, 2, 3, 4, 5, and 7 in the *WNT5A* intron 1 sequence (*P* < 0.05; Figure 8A). Synergistic action of E_2_ and PGE_2_ resulted in hypermethylation of CpGs at positions 1, 2, 3, and 4 in the *ADH1C* intron 3 sequence (*P* < 0.05; Figure 3A) gene and CpT at position 1 in the *PSAT* exon 1 sequence (*P* < 0.05; Figure 6A).

Studying the DNA methylation levels of selected gene sequences in endometrial samples collected from the gilts on day 12 of pregnancy, we found a hypermethylation of CpG at position 4 in the *ADAMTS20* intron 35 sequence (*P* < 0.05; Figure 2B) and in CpGs at positions 2, 3, 4, 7, and 8 in the *BGN* intron 1 sequence (*P* < 0.05; Figure 4B) when compared to endometrial samples collected from the gilts on day 12 of the estrous cycle. Hypomethylation of CpGs at positions 3, 4, 5, 6, and 7 of the *DMBT1* intron 24 sequence (*P* < 0.05; Figure 5B); CpGs at positions 4 and 9 of the *RASSF1* promoter region sequence (*P* < 0.05; Figure 7B); and CpG at position 8 in the *WNT5A* intron 1 sequence (*P* < 0.05; Figure 8B) were found in DNA isolated from endometrial samples collected on day 12 of pregnancy when compared to endometrial samples collected from the gilts on day 12 of the estrous cycle.

### E2 and/or PGE2 and the presence of conceptuses regulate endometrial gene expression

Using an in vivo model of intrauterine hormonal infusions, we evaluated the effect of E_2_, PGE_2_, and E_2_ + PGE_2_ on the endometrial expression of selected genes. We found the effect of treatment (*P* < 0.05) and the effect the site of hormone administration (*P* < 0.05) on *ADAMTS20* mRNA expression in porcine endometrium (Figure 2C), and only the effect of treatment (*P* < 0.05) on endometrial mRNA levels of *ADH1C*, *BGN*, *DMBT1*, *PSAT1*, *RASSF1*, and *WNT5A* (Figures 3C, 4C, 5C, 6C, 7C, and 8C, respectively). We also found the effect of reproductive status (cyclic vs. pregnant) on endometrial mRNA expression of *ADAMTS20, ADH1C*, *BGN*, *DMBT1*, *PSAT1*, and *WNT5A* (Figures 2D, 3D, 4C, 5C, 6C, and 8C, respectively)*.*

E_2__833 ng administered alone increased the endometrial mRNA expression of *ADH1C* and *DMBT1* and decreased the endometrial abundance of *WNT5A* transcript (*P* < 0.05, Figures 3C, 5C, and 8C, respectively). Intrauterine infusions of E_2__33.3 μg increased the endometrial mRNA expression of *ADAMTS20* in the hormone-treated horn (*P* < 0.05, Figure 2C) and the *DMBT1* (*P* < 0.05, Figure 5C) mRNA in both placebo- and hormone-treated horns but decreased the mRNA levels of *PSAT1*, *RASSF1*, and *WNT5A* in placebo and hormone-treated horns (*P* < 0.05, Figures 6C, 7C, and 8C, respectively). PGE_2_ administered alone into the uterine lumen increased the endometrial mRNA expression of *BGN* (*P* < 0.05; Figure 4C) but decreased the expression of *WNT5A* (*P* < 0.05, Figure 8C). Administration of E_2_ + PGE_2_ resulted in increased levels of *DMBT1* mRNA in the porcine endometrium (*P* < 0.05, Figure 5C) but decreased the endometrial abundance of *PSAT1*, *RASSF1*, and *WNT5A* transcripts (*P* < 0.05; Figures 6C, 7C, and 8C, respectively).

In endometrial samples collected from the gilts on day 12 of pregnancy, we observed an increased mRNA expression of *ADAMTS20*, *ADH1C*, *BGN*, *DMBT1* (*P* < 0.05, Figures 2D, 3D, 4D, and 5D, respectively) and decreased expression of *PSAT1* and *WNT5A* mRNAs (*P* < 0.05; Figures 6D and 8D) when compared to the endometrial samples from the gilts on day 12 of the estrous cycle.

### Studying the correlation between changes in CpG methylation levels and altered expression of selected genes in the porcine endometrium

Studying the correlation between changes in the methylation levels of single CpG sites in DNA sequences of selected differentially expressed genes, we found a significant, positive correlation (*P* = 0.0275, *r* = 0.72) between the hypermethylated CpG at position 4 in the *ADAMTS20* intron 35 sequence in response to pregnancy and mRNA expression of *ADAMTS20* in endometrial samples collected from the gilts on day 12 of pregnancy when compared to day 12 of the estrous cycle (Supplementary Figure 3A).

A significant positive correlation was found between the hypermethylated CpG at position 2 in the *ADH1C* intron 3 sequence and the mRNA levels of *ADH1C* in the endometrial samples collected from E_2__833 ng-treated the gilts (*P* = 0.022, *r* = 0.78, Supplementary Figure 3B).

A strong positive correlation was detected for hypermethylated CpGs at positions 2 (*P* = 0.0067, *r* = 0.89), 3 (*P* = 0.0319, *r* = 0.80), 4 (*P* = 0.0046, *r* = 0.91), 7 (*P* = 0.0081, *r* = 0.89), and 8 (*P* = 0.0399, *r* = 0.83) in the *BGN* intron 1 sequence and *BGN* mRNA expression in the endometrial samples collected from the gilts on day 12 of pregnancy when compared to day 12 of the estrous cycle (Supplementary Figure 3C–G).

A negative correlation was detected for hypomethylated CpGs at positions 3 (*P* = 0.054, *r* = −0.87), 4 (*P* = 0.0412, *r* = −0.73), 5 (*P* = 0.0166, *r* = −0.76), 6 (*P* = 0.0008, *r* = −0.90), and 7 (*P* = 0.0099, *r* = −0.80) in the sequence of intron 24 of the *DMBT1* gene and its mRNA abundance in endometrial samples collected from the gilts on day 12 of pregnancy when compared to day 12 of the estrous cycle (Supplementary Figure 3H–L).

A significant negative correlation (*P* = 0.0166, *r* = −0.76) was detected for hypermethylated CpT at position 1 in the *PSAT1* exon 1 sequence and mRNA levels of *PSAT1* in endometrial samples collected from E_2__33.3 μg-treated gilts compared to the gilts from the control group (Supplementary Figure 3M).

Positive correlations were found for hypomethylated CpGs at positions 1 (*P* = 0.0195, *r* = 0.66), 5 (*P* = 0.0337, *r* = 0.61), and 7 (*P* = 0.0096, *r* = 0.71) in the *WNT5A* intron 1 sequence and its mRNA expression in E_2__33.3 μg-treated gilts when compared to the gilts from the control group (Supplementary Figure 3N–P). Similarly, a significant positive correlation (*P* = 0.0275, 0.66) was detected for the hypomethylated CpG at position 7 in intron 1 of *WNT5A* and its mRNA levels in the endometrial samples collected from the gilts treated with E_2_ + PGE_2_ when compared to the control gilts (Supplementary Figure 3Q). Moreover, a strong positive correlation (*P* = 0.0008, *r* = 0.93) was detected between the hypomethylated CpG at position 8 of *WNT5A* intron 1 and its mRNA expression in endometrial samples collected from the gilts on day 12 of pregnancy when compared to day 12 of the estrous cycle (Supplementary Figure 3R).

## Discussion

Molecular interactions occurring in the endometrium during early pregnancy in mammals including pigs are essential for understanding the complex mechanisms responsible for successful maternal recognition of pregnancy, implantation, and development of embryos. In our present report, we applied an in vivo model of hormone infusions into the uterine lumen reflecting conceptus signaling in order to study their involvement in the regulation of gene expression by affecting endometrial DNA methylation. Herein, we provide new insights into mechanisms of differential gene expression by DNA-methylation changes in the porcine endometrium in response to embryonic signals, particularly E_2_ and/or PGE_2_.

Studies on mechanisms involved in tumor etiology indicate a significant role of E_2_ in epigenetic processes related to DNA methylation and histone modifications [16]. The process of DNA methylation is catalyzed by DNA methyltransferases: DNMT1, DNMT3A, and DNMT3B [19, 20]. Our present results indicate that E_2_ affected mRNA and protein expression of DNMT in the porcine endometrium in response to E_2_, PGE_2_, E_2_ + PGE_2_, and in response to pregnancy in a similar manner. Intriguingly, we found that intrauterine infusion of E_2_ led to decreased mRNA expression of *DNMT1*, *DNMT3A*, and *DNMT3B*. The protein level of DNMT1 and DNMT3A was decreased by the E_2_ + PGE_2_ treatment, whereas the expression of the DNMT3B protein was decreased both by E_2_ acting alone and by E_2_ + PGE_2_. Likewise, decreased expression of DNMT1 and DNMT3A proteins was also observed in the endometrial samples collected from the gilts on day 12 of pregnancy. These results are in line with previous studies demonstrating that E_2_ regulated DNMT mRNA and protein expression in human epithelial cell lines of tumor origin and in other tissues in vivo, such as the dorsal hippocampus [39–41]. Our findings are also consistent with a report indicating that the expression of DNMTs in the human endometrium decreased during the secretory phase and was reduced by E_2_ and P4 in endometrial stromal cells [42]. Similarly, our recent findings indicate that E_2_ controlled DNMT expression in the porcine corpus luteum in vivo and changes in their abundance were significant during pregnancy [31]. Interestingly, recent studies have indicated that E_2_ can affect the expression of DNMTs via a complex mechanism involving a number of co-regulators [43]. The divergences between mRNA and protein levels for DNMT1 in response to treatments may be related to different regulatory mechanisms controlling transcription and translation processes as described earlier [44]. A time shift between mRNA and protein synthesis might likewise be the underlying cause.

We observed the effect of hormones administered to one uterine horn also in uterine horns which received placebo infusion in the hormone-treated groups. Embryos may not only affect the uterine horn in which they are present by secreting signals as direct local action, also called as “local effect,” but also embryonic signals can be transferred by local blood and lymph circulation systems to the adjacent uterine horns and ovaries and affect the gene and protein expression there as indirect local action, also called as “systemic effect” [28, 45]. By using our model in which we administered hormones only to one randomly selected uterine horn and placebo to the adjacent uterine horn, we were able to assess the direct local and indirect local effects of E_2_ and PGE_2_ on the endometrial gene and protein expression and DNA methylation.

The present results demonstrated that estradiol treatment of porcine uteri in vivo led to increased activity of DNMTs in the endometrial samples in contrast to decreased mRNA expression of DNMTs. Moreover, the activity of DNMTs was increased in endometrial samples collected from the gilts on day 12 of pregnancy when compared to day 12 of the estrous cycle. Our results are consistent with studies on the invasive type of implantation, which proved an important role of DNMT in preparing mouse endometrium for implantation [46, 47], also indicating that inhibition of DNMT activity resulted in decreased expression of DNA methyltransferases and essential endometrial genes, which in turn, reduced embryo implantation [47]. Interestingly, in contrast to our results, it has been reported that E_2_ elevated the expression of the DNMT3B mRNA and protein. However, in agreement with our findings, E_2_ stimulated the activity of DMNT in human endometrial adenocarcinoma cell line (Ishikawa cell line) [18]. The discrepancy between the results of DNMT expression and activity in the endometrium could be a result of the same study time point for DNMT mRNA/protein expression on the one hand and DNMT activity on the other. It is possible that the increased expression of DNMT could have occurred earlier during the E_2_ treatment than the increased activity of these enzymes. Moreover, the fact that E_2_ stimulated the expression of DNMTs in tumor tissue does not necessarily imply the same effect under physiological conditions. Unlike in tumors, all physiological processes occurring are tightly controlled. Therefore, it is possible that the stimulating effect of E_2_ and E_2_ + PGE_2_ on DNMT mRNA/protein expression may be attenuated by other factors of endometrial and/or conceptus origin. Moreover, subtle effects of E_2_ and/or PGE_2_ acting on endometrial *DNMT1, DNMT3A, DNMT3B* mRNA abundance as well as on DNMT3B protein expression in porcine endometrium could be due to the using doses of hormones administered into uterine lumen close to physiological levels. Therefore, the physiological tissue response could be not as prominent as in cases when much higher doses of hormones are applied or in pathological conditions.

Once we established that E_2_ and E_2_ + PGE_2_ affected the expression and activity of enzymes involved in DNA methylation, the question arose whether E_2_ per se can induce changes in DNA methylation levels. Since whole genome bisulfite sequencing is a comprehensive but relatively expensive analysis, we decided to precede with initial gene-specific studies by pyrosequencing. We hereby selected sequences rich in CpG sites localized in promoter regions or gene bodies of genes involved in processes important for pregnancy establishment. Based on our recent results from endometrial transcriptome profiling [4, 25] and studies on the effect of short-term exposure to E_2_ on various tissues in pigs [26, 35], we selected the seven candidate genes *ADAMTS20*, *ADH1C*, *BGN*, *DMBT1*, *PSAT1*, *RASSF1*, and *WNT5A*, which we knew that their endometrial expression was altered either in response to hormonal treatment in vivo or to pregnancy. The selected genes are important for processes accompanying development of early pregnancy such as metabolism (*ADH1C*), cell cycle regulation (*PSAT1*), cell proliferation (*RASFF1* and *DMBT1*), and tissue remodeling (*ADAMTS20*, *BGN,* and *WNT5A*) [4, 25, 26, 35]. Interestingly, only for sequences localized in the intron 3 of *ADH1C* and in the exon 1 of *PSAT1* did we detect a significant effect of E_2_ and/or E_2_ + PGE_2_ resulting in hypermethylation of single CpG sites in porcine endometrial samples. An analysis of sequences localized in the gene body of *ADAMTS20*, *BGN*, and *WNT5A* genes revealed hypomethylation of single CpG sites following E_2_ and E_2_ + PGE_2_-treated gilts. Intriguingly, the same sequences analyzed in endometrial tissues collected from the gilts on day 12 of pregnancy and the estrous cycle displayed reversed patterns of CpG site methylation. In endometrial samples collected from the gilts on day 12 of pregnancy, hypermethylation of single CpGs was observed in intronic sequences of *ADAMTS20* and *BGN*, whereas hypomethylation of single CpG sites was observed in the intronic sequences of *DMBT1* and *WNT5A* and in the promoter region of *RASSF1* gene.

The importance of DNA methylation in the regulation of gene expression involved in reproductive functions has also been described for other species [48–50]. However, compared to tumors, the effect of hormones at physiological doses on DNA methylation observed in this study was more modest. Our findings correspond to the previous reports on subtle changes in single CpN-site methylation in porcine prostate and endometrium in response to E_2_ treatment [26, 35]. Likewise, only a weak effect of an oral E_2_ treatment was detected on the hypermethylation of one CpG site within the *HOXA10* promoter sequence in porcine uteri collected from prepubertal piglets [51].

Initially, the general paradigm assumed that methylation occurring in the promoter region or in the transcription binding sites of a gene results in repressed transcriptional activity [52]. However, recent studies have revealed that this mechanism is more complex, since methylation has been shown to elicit site-specific effects. Indeed, methylation may block transcription in the transcription starting sites; however, it may also promote transcription when occurring in the gene body not only in exons but also in intron regions [53–55]. In order to determine whether changes in methylation levels of single CpG sites localized in selected gene sequences may be related to their altered mRNA abundance, we correlated the values of the single CpG methylation levels with the values of the mRNA levels. A positive correlation between E_2_-affected hypermethylation of single CpG sites localized in intronic sequences with increased gene expression was found for *ADH1C* and *WNT5A*. Interestingly, E_2_-affected hypermethylation of CpT at position 1 in the *PSAT1* exon 1 was found to be negatively correlated with the increased *PSAT1* endometrial mRNA expression. By studying the correlation of differentially methylated single CpG sites and mRNA expression in endometrial samples collected on day 12 of pregnancy and the estrous cycle, we found that a hypomethylation of single CpG sites detected in endometrial samples collected from pregnant gilts was positively correlated with increased mRNA expression of *ADAMTS20*, *BGN*, and *WNT5A*. In contrast, decreased methylation of CpG sites in *DMBT1* intron 24 sequence was negatively correlated with its increased expression in porcine endometrium. We found both positive and negative correlations between CpG sites methylation of intronic regions and endometrial gene expression that correspond with previous reports [55, 56]. Interestingly, it has been evidenced that DNA methylation found in intronic regions correlates positively or negatively with gene expression in heart tissue depending either on physiological or pathological state [56]. On the other hand, it should be noted that even a significant correlation between changes in DNA methylation and the expression of analyzed genes does not necessarily confirm dependency between these two processes. Further studies should assess the effect of targeted methylation of selected DNA sequences on the expression of corresponding genes.

Thus, our results confirm that there was no universal and direct dependency between differences in methylation levels of single CpG sites and mRNA expression. To determine whether hyper- or hypomethylation on a global scale resulted in changes to mRNA expression, a more sophisticated approach (e.g., site-specific methylation studies) is required. Moreover, different patterns of CpG methylation detected in hormone-treated and cyclic pregnant animals suggest that other unidentified factors may contribute to the establishment of a final methylation status in a particular physiological state. In the present study, we used whole tissue fragments. Therefore, some potential cell-specific changes could be missed. On the other hand, pyrosequencing as the gold standard methylation quantification method for single CpG sites allowed us to study short, selected sequences to impede our understanding of epigenetic events occurring in porcine endometrium during early pregnancy. It is likely that the detected changes were not the only ones that occurred due to the different treatments and trials. Therefore, further studies involving high-throughput sequencing need to be conducted.

In summary, we identified the effect of the major embryonic signals E_2_ and PGE_2_ as well as the conceptus on epigenetic processes in the porcine endometrium during the period of pregnancy establishment. We detected the methylation changes of single CpG sites within a selected gene DNA sequence, which in part correlated with their altered mRNA expression. Our results indicate a novel role for embryo signaling in the regulation of DNA methylation as putative physiological mechanisms controlling endometrial gene expression during pregnancy establishment.

## Data availability

All data are incorporated into the article and its online supplementary material.

## Author contributions

PK—performing most experiments, data analysis and interpretation, contribution to study design, data mining and presentation, writing the draft of the manuscript, and incorporation revisions. VW—optimization of pyrosequencing experiments, data interpretation, and revision of the manuscript. EG and MB—contributed to gene expression analyses, revision of the manuscript. SU—contributed to conceptualization of pyrosequencing studies, their methodology, data interpretation, and critical revision of the manuscript. AW—conceptualization, supervision, and optimization of experiments; data analysis and interpretation; editing and critical revision of the manuscript text; project management; funding acquisition.

## Conflict of interest

The authors declare no competing interests.

## Ethics declaration

The use of animals was in accordance with the Act of 15th of January 2015 on the Protection of Animals Used for Scientific or Educational Purposes and Directive 2010/63/EU of the European Parliament and the Council of 22nd of September 2010 on the protection of animals used for scientific purposes. Experiment 1 was conducted in accordance with the national guidelines for agricultural animal care and was approved by the Animal Ethics Committee, University of Warmia and Mazury in Olsztyn, Poland, permission No. 17/2008. Tissues used in experiment 2 were collected from animals bound for commercial slaughter and meat production.

## Supplementary Material

SUPPLEMENTARY_FIGURE_1_ioac193

Supplementary_Figure_2A_revised_ioac193

Supplementary_Figure_2B_ioac193

Supplementary_Figure_2C_revised_ioac193

Supplementary_Figure_3_A-G_ioac193

Supplementary_Figure_3_H-M_ioac193

Supplementary_Figure_3_N-R_ioac193

Supplementary_Information_07_09_16_10_ioac193

Supplementary_Table_1_ioac193

Supplementary_Table_2_revised_ioac193

Supplementary_Table_3_ioac193

SUPPLEMENTARY_FIGURE_LEGENDS_ioac193
