# Supplementary figures and images for: Novel role for conceptus signals in mRNA expression regulation by DNA methylation in porcine endometrium during early pregnancy
Source: Biol Reprod. 2022 Nov 2;108(1):150–68. doi: 10.1093/biolre/ioac193 (PMC9843678; doi:10.1093/biolre/ioac193)

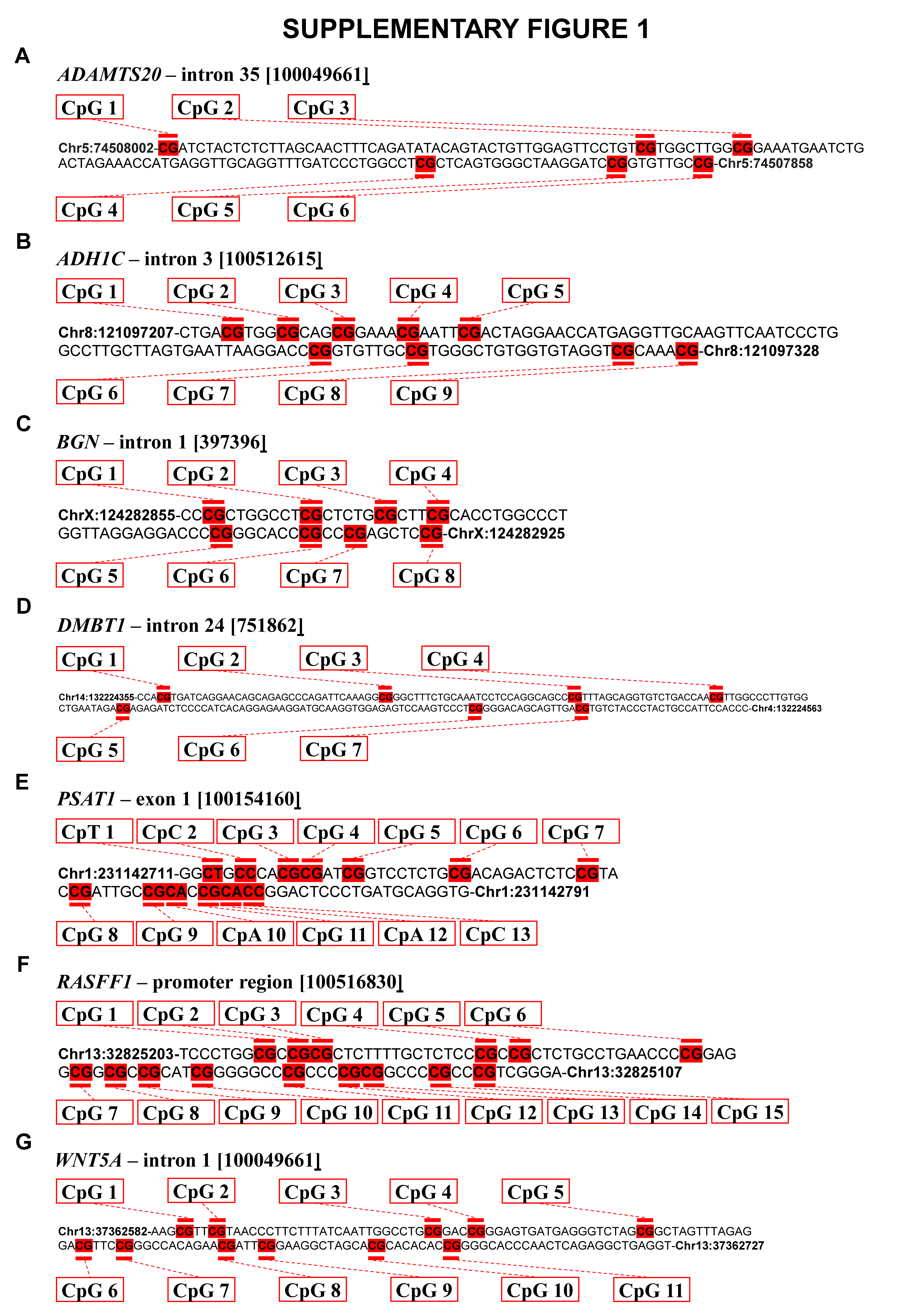

Supplement: SUPPLEMENTARY_FIGURE_1_ioac193 [file supplementary_figure_1_ioac193.zip › SUPPLEMENTARY_FIGURE_1_ioac193.tif]

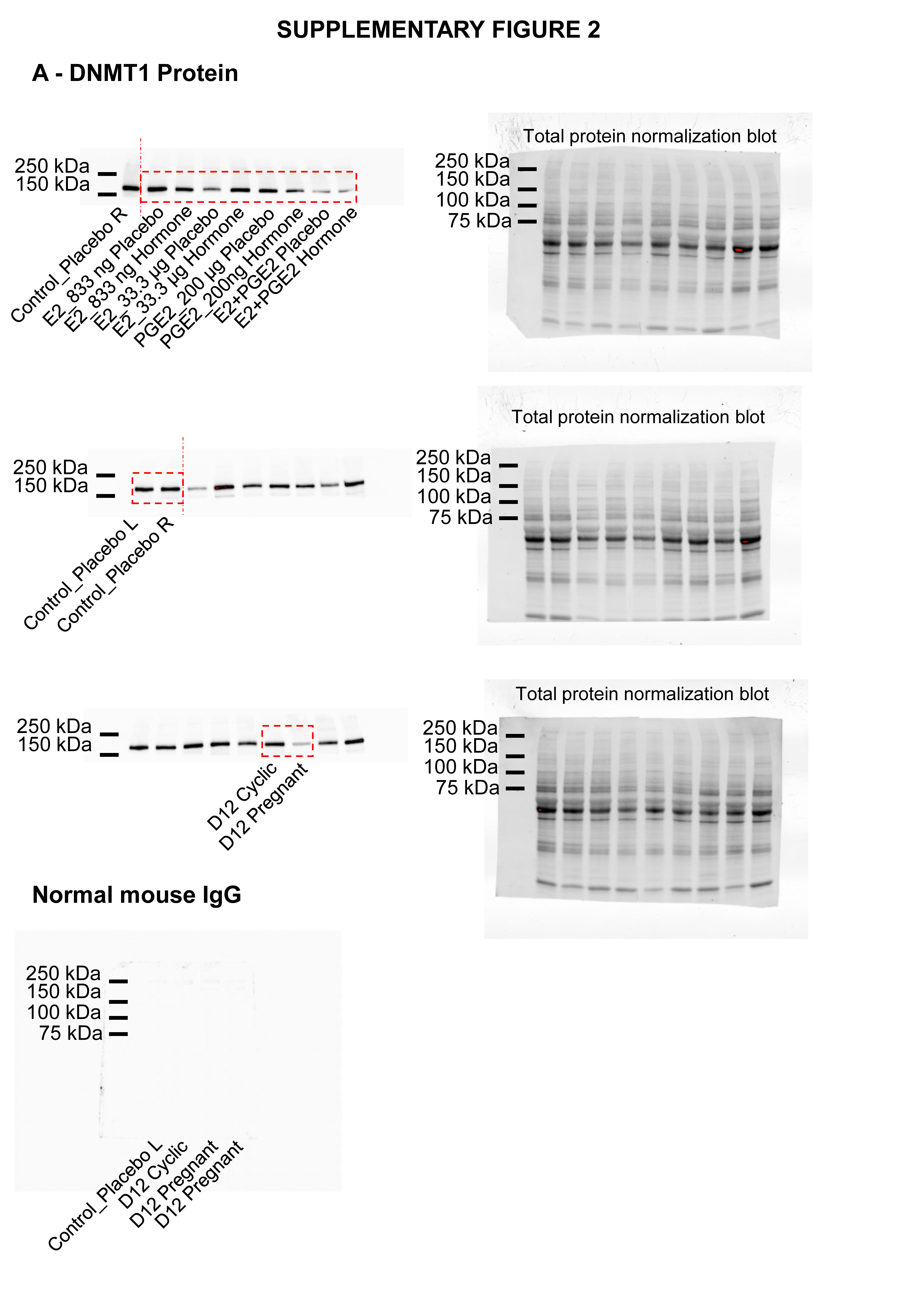

Supplement: Supplementary_Figure_2A_revised_ioac193 [file supplementary_figure_2a_revised_ioac193.zip › Supplementary_Figure_2A_revised_ioac193.tif]

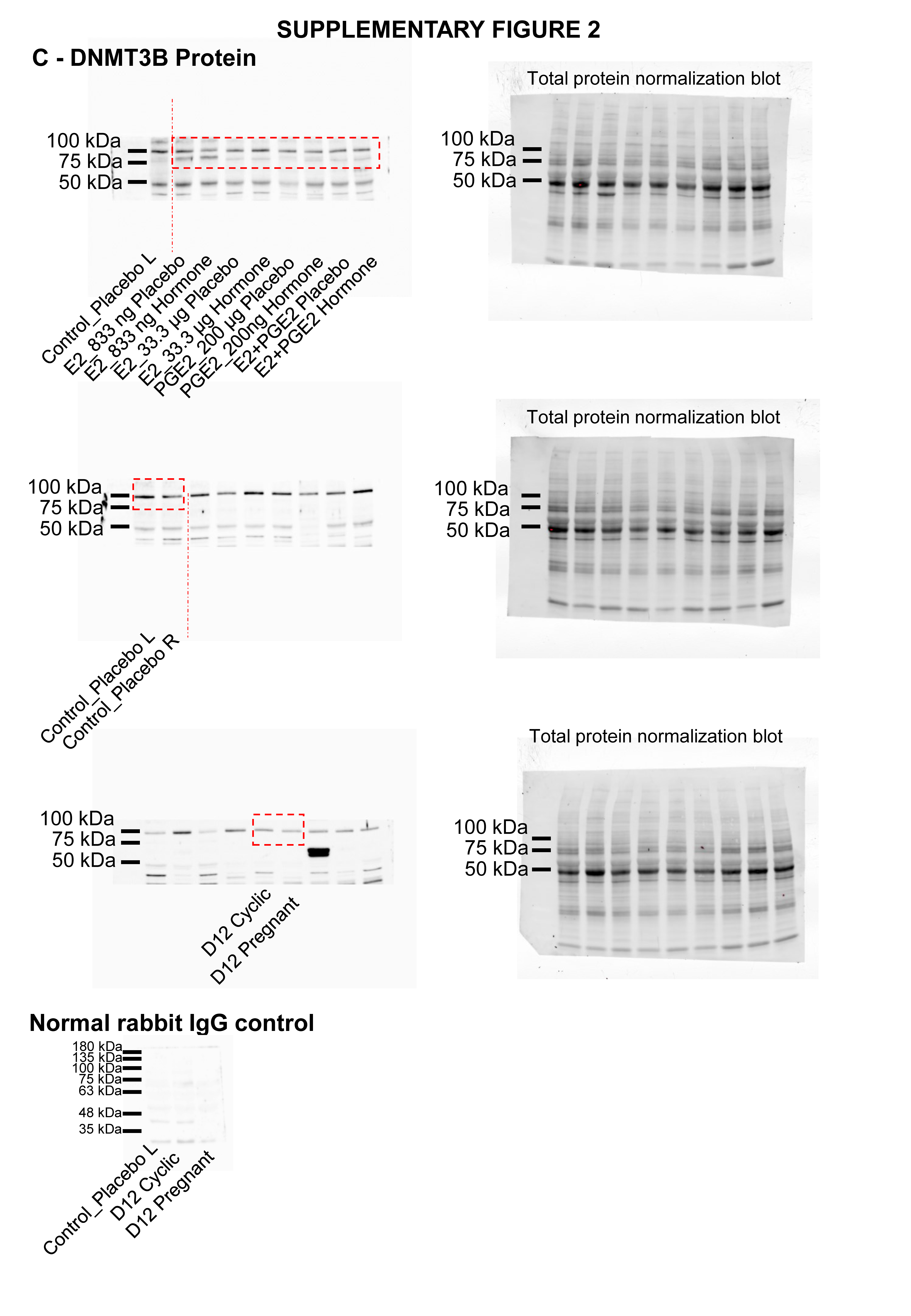

Supplement: Supplementary_Figure_2C_revised_ioac193 [file supplementary_figure_2c_revised_ioac193.zip › Supplementary_Figure_2C_revised_ioac193.tif]

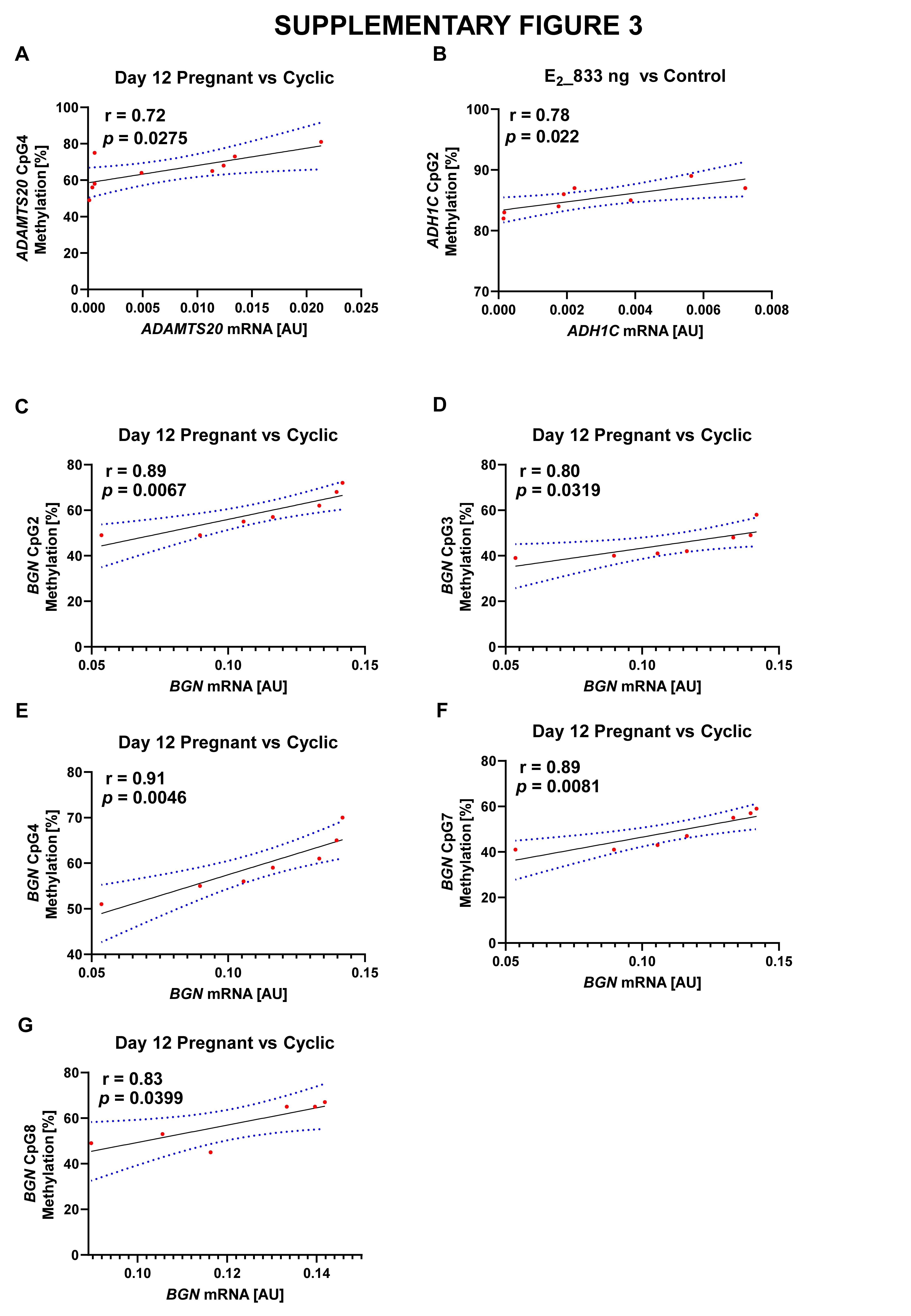

Supplement: Supplementary_Figure_3_A-G_ioac193 [file supplementary_figure_3_a-g_ioac193.jpeg]

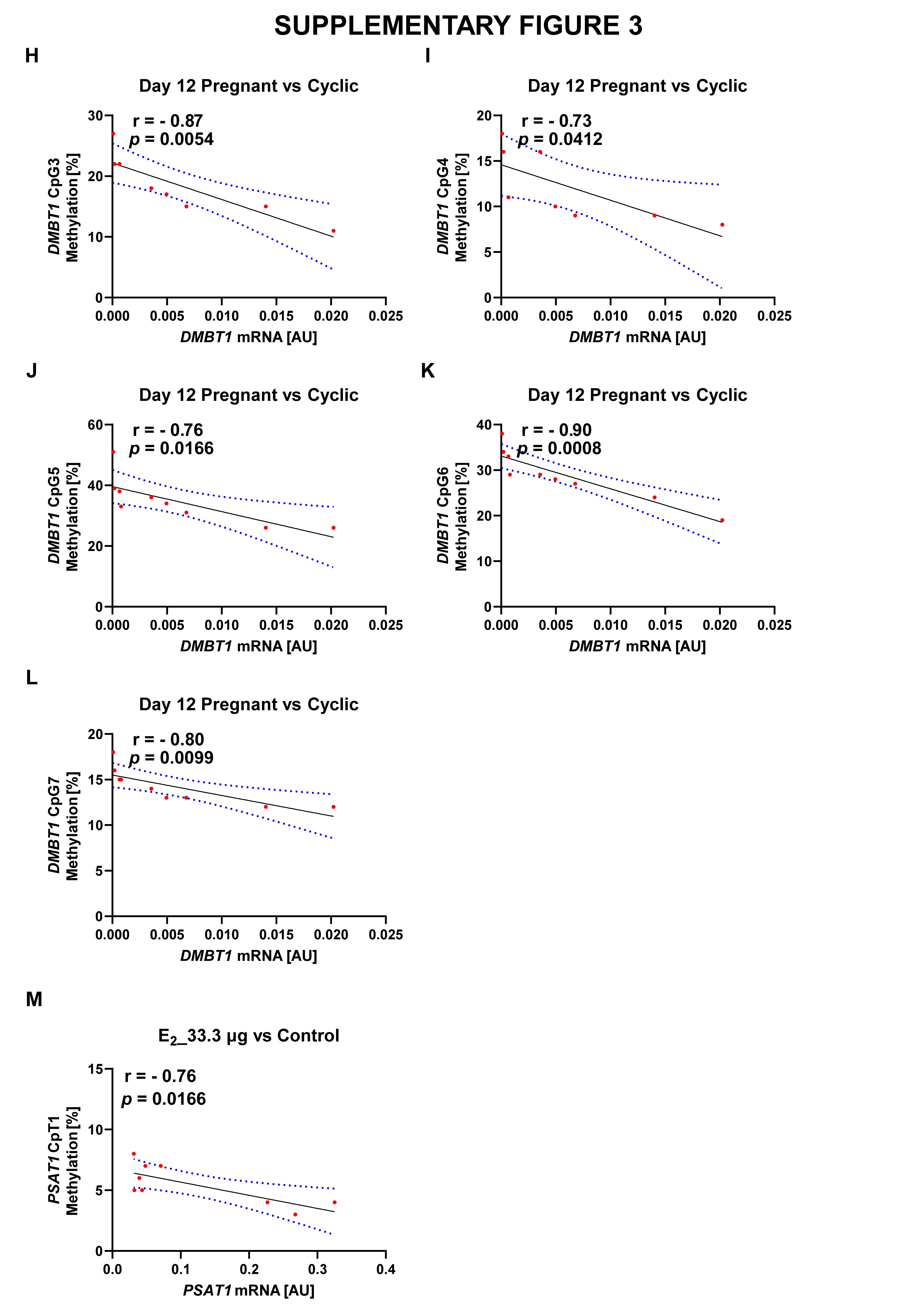

Supplement: Supplementary_Figure_3_H-M_ioac193 [file supplementary_figure_3_h-m_ioac193.jpeg]

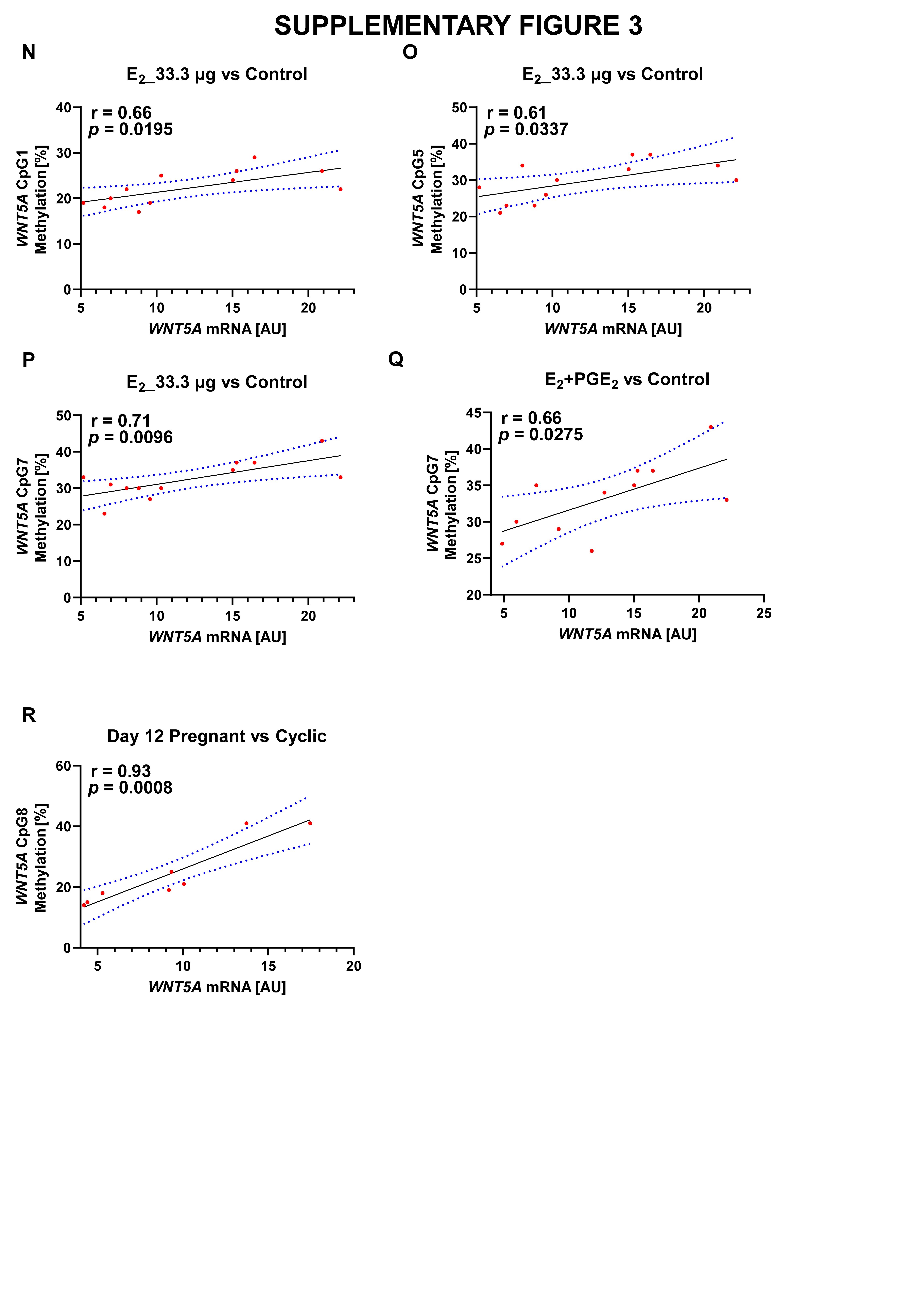

Supplement: Supplementary_Figure_3_N-R_ioac193 [file supplementary_figure_3_n-r_ioac193.jpeg]
