# Supplementary material for: Novel role for conceptus signals in mRNA expression regulation by DNA methylation in porcine endometrium during early pregnancy
Source: Biol Reprod. 2022 Nov 2;108(1):150–68. doi: 10.1093/biolre/ioac193 (PMC9843678; doi:10.1093/biolre/ioac193)
Supplement: Supplementary_Information_07_09_16_10_ioac193 [file supplementary_information_07_09_16_10_ioac193.docx]

**SUPPLEMENATRY INFORMATION**

*Gene expression studies*

To study endometrial expression of *DNMT1*, *DNMT3A* and *DNMT3B* genes, total RNA was isolated from collected endometrial samples with the Total RNA mini kit (A&A Biotechnology, Gdansk, Poland) accordingly to the manufacturer’s protocol. The quality and quantity of isolated RNA was evaluated by electrophoresis and by using the NanoDrop 1000 spectrophotometer (Thermo Fisher Scientific Inc; DE; USA). Equal amounts of isolated RNA (1 µg) were treated with DNase I (Life Technology Inc.; Carlsbad, CA, USA) according to the manufacturer’s protocol to exclude possible DNA contamination. Dnase-treated RNA was used to generate cDNA template for Real Time PCR reaction. Dnase-treated RNA was reverse transcribed using the MultiScribe™ Reverse Transcriptase kit (Life Technologies; Carlsbad; CA; USA) according to the manufacturers protocol. cDNA samples were stored in -80 °C for further Real-Time RT-PCR analyses. Real-Time RT-PCR for *DNMT1*, *DNMT3A* and *DNMT3B* genes was performed with the Applied Biosystems 7900 Real-Time PCR system (Life Technologies) using specific primers (Supplemental Table 1) and Power SYBR Green master mix (Life Technologies). Reverse transcribed cDNA was amplificated in 25 µl of reaction mixture containing 12.5 µl Power SYBR Green master mix, 2.5 µl of each sense and antisense primer (1 µM), 3.5 µl of cDNA and 4 µl of water. The PCR programs for analyzed genes were performed as follows: initial denaturation (95 °C; 15 min) followed by 40 cycles of denaturation (95 °C for 15 seconds), annealing and elongation (60 °C for 1 minute). For *ACTB*, *PPIA*, *GAPDH* and *RPL13A* genes the PCR program was: initial denaturation (95 °C; 15 min) followed by 36 cycles of denaturation (95 °C for 15 seconds), annealing (55 °C for 30 seconds) and elongation (72 °C for 1 minute). After the PCR, melting curves were acquired by gradual increases in the temperature from 60 to 95 °C to ensure that a single product was amplified in the PCR reaction. Expression values were calculated by using PCR Miner software [1]. Stability of the reference genes (*ACTB*, *PPIA*, *GAPDH* and *RPL13A)* was assessed using the statistical algorithms NormFinder 2.0 [2]. The most stable was the geometrical mean of *GAPDH* and *RPL13A* expression values.

To determine the abundance of *ADH1C*, *BGN*, *PSAT1* and *RASSF1* mRNAs in endometrial samples, isolated total RNA (1 µg) was treated with DNase I (Life Technologies) and reverse transcribed using MultiScribe™ Reverse Transcriptase kit (Life Technologies) accordingly to the manufacturer’s protocol. Synthesized cDNA was used in qPCR reactions. Real-Time PCR for *ADH1C*, *BGN*, *PSAT1*, *RASSF1, ACTB*, *PPIA*, *GAPDH* and *RPL13A* genes was performed with the Applied Biosystems 7900 Real-Time PCR system (Life Technologies) using TaqMan assays (Thermo Fisher Scientific; Supplemental Table 1) accordingly to the manufacturer’s protocol. The PCR program was: initial denaturation (95 °C; 15 min) followed by 36 cycles of denaturation (95 °C for 15 seconds), annealing and elongation (60 °C for 1 minute). Expression values were calculated using the PCR Miner software [1]. Stability of the reference genes (*ACTB*, *PPIA*, *GAPDH* and *RPL13A)* was assessed using the statistical algorithm NormFinder 2.0 [2]. The geometrical mean of *GAPDH* and *RPL13A* expression values was most stable.

**REFERENCES**

1. Zhao S, Fernald RD. Comprehensive algorithm for quantitative real-time polymerase chain reaction. J Comput Biol 2005; 12:1047-1064.

2. Andersen CL, Jensen JL, Orntoft TF. Normalization of real-time quantitative reverse transcription-PCR data: a model-based variance estimation approach to identify genes suited for normalization, applied to bladder and colon cancer data sets. Cancer Res 2004; 64:5245-5250.
