# Supplementary material for: Novel role for conceptus signals in mRNA expression regulation by DNA methylation in porcine endometrium during early pregnancy
Source: Biol Reprod. 2022 Nov 2;108(1):150–68. doi: 10.1093/biolre/ioac193 (PMC9843678; doi:10.1093/biolre/ioac193)
Supplement: Supplementary_Table_1_ioac193 [file supplementary_table_1_ioac193.docx]

| **Supplementary Table 1. -TaqMan assays and primer sequences used in qPCR analyses.** | | | |
| --- | --- | --- | --- |
| **Gene symbol** | **TaqMan assay ID / primer sequence** | **RefSeq ID / GenBank accession no.** |  |
| *ADAMTS20* | Ss03372950_g1 | NM_001257275.1 |  |
| *ADH1C* | Ss04325972_m1 | NM_001243939.1 | |
| *BGN* | Ss03375454_u1 | XM_003135475.5 | |
| *DMBT1* | Ss03380964_u1 | NM_001048188.1 | |
| *GAPDH* | Ss03373286_u1 | NM_001206359.1 | |
| *PPIA* | Ss03394782_g1 | NM_214353.1 | |
| *PSAP* | Ss04248211_m1 | NM_001198919.1 | |
| *PSAT1* | Ss06878864_m1 | XM_021065150.1 | |
| *RASSF1* | Ss06887894_m1 | XM_003358449.3 | |
| *RPL13A* | Ss03376908_u1 | NM_001244068.1 | |
| *WNT5A* | Sense:5’-TCTCCTTCGCCCAGGTTGTA-3’  Antisense:5'-GGCTGTGCTCCTATGATATATACTTCTG-3’ | NC_010455.5 | |
| *GAPDH* | Sense:5’-CAGCAATGCCTCCTGTACCA-3’ Antisense:5-GATGCCGAAGTTGTCATGGA-3’ | NM_001206359.1 | |
| *RPL13A* | Sense:5'-AGTTAAAGTACCTGGCCTTCCT-3' Antisense:5'-TGGCCTCTCTTGGTCTTG-3' | NM_001244068.1 | |
