# Supplementary material for: Novel role for conceptus signals in mRNA expression regulation by DNA methylation in porcine endometrium during early pregnancy
Source: Biol Reprod. 2022 Nov 2;108(1):150–68. doi: 10.1093/biolre/ioac193 (PMC9843678; doi:10.1093/biolre/ioac193)
Supplement: Supplementary_Table_2_revised_ioac193 [file supplementary_table_2_revised_ioac193.docx]

**Supplementary table 2 - List of antibodies and controls used in Western Blot analyses.**

| **Peptide/Protein Target** | **Antigen Sequence** | **Name of Antibody** | **Manufacturer, Catalog No., or Name of Source** | **Species Raised in Monoclonal or Polyclonal** | **Dilution used** |
| --- | --- | --- | --- | --- | --- |
| DNMT1 | EKDDREDKENAFKR | DNMT1 Antibody | NB100-56519  Novus Biologicals | Mouse, monoclonal | 1:1000 |
| DNMT3A | N/A | DNMT3A (D23G1) Rabbit mAb | #3598  Cell Signaling Technology | Rabbit, monoclonal | 1:300 |
| DNMT3B | RGRRSSSRLSKREVSSC | DNMT3B Antibody | orb372330  Biorbyt | Rabbit, polyclonal | 1:100 |
| Anti-mouse, secondary antibodies | N/A | Immun-Star Goat Anti-Mouse (GAM)-HRP Conjugate | Bio-Rad; 1705047 | Goat, polyclonal | 1:20 000 |
| Anti-rabbit, secondary antibodies | N/A | Immun-Star Goat Anti-Rabbit (GAM)-HRP Conjugate | Bio-Rad; 1706515 | Goat, poyclonal | 1:20 000 |
| Rabbit Control IgG | N/A | Normal rabbit  IgG | Santa Cruz SC-2027 X | Rabbit | 1:1250^*^ |
| Mouse Control  IgG | N/A | Normal mouse  IgG | Abcam  ab37355 | Mouse | 1:5000^*^ |

^*^ - Concentration of isotype control was calculated to match the protein concentration of primary antibodies
