# Supplementary material for: Novel role for conceptus signals in mRNA expression regulation by DNA methylation in porcine endometrium during early pregnancy
Source: Biol Reprod. 2022 Nov 2;108(1):150–68. doi: 10.1093/biolre/ioac193 (PMC9843678; doi:10.1093/biolre/ioac193)
Supplement: Supplementary_Table_3_ioac193 [file supplementary_table_3_ioac193.docx]

| **Supplementary Table 3 - Primers used for methylation analysis of bisulfite converted DNA.  All reverse primers were biotinylated. AT - annealing temperature.** | | | | | | |
| --- | --- | --- | --- | --- | --- | --- |
| **Assay ID** | **Sequence 5` to 3`** | **Orientation** | **Product size [bp]** | **AT [° C]** | **Sequencing primer 5` to 3`** |  |
| *ADAMTS20* - intron 35 | GTAGTATAAAGTAATTATTTGGTTGAGTATT | FWD | 286 | 55 | TTTTTTGTGATGAGAAT |  |
|  | ATCTATAACCCACACCAAACTC | REV |  |  |  |  |
| *ADH1C* - intron 3 | TGTAAGGTTTTGAGGTAATAAAAGTAAAT | FWD | 264 | 57.5 | ATTAATAGTAGATTTTAGGAGTTT |  |
|  | AACCATAACAATACCAAATTCAAACC | REV |  |  |  |  |
| *BGN* - intron 1 | TAGGTTGGGTGGTTGGTAT | FWD | 260 | 57 | GGTTTTTTTTTTTGATTGTTAGT |  |
|  | AAATTTCCTTCCACACCCTATTCCT | REV |  |  |  |  |
| *DMBT1* - intron 24 | TGGAGGTTTATAGGGTTAAGTTATTTGT | FWD | 258 | 51 | TAAGTTATTTGTTTAGGG |  |
|  | TACCAAAAACACTCAAAATAAAATAACAA | REV |  |  |  |  |
| *PSAT1* - exon1 | GAGTAGGAGTTGGTTGTAAGAT | FWD | 120 | 58 | AGTTGGTTGTAAGATTTG |  |
|  | ACCCCCAAATTAACCACCTACATCAA | REV |  |  |  |  |
| *RASSF1* - promoter region | AGGGATGAAGGGAGGTAG | FWD | 244 | 56 | TTTGGGTGTTGGGTA |  |
|  | ACCCAAACTCCTACTAACTCTAAAC | REV |  |  |  |  |
| *WNT5A* - intron 1 | AGGAGGTGAGGGTAAAGTGGTTTTATA | FWD | 258 | 57 | GAGTTTTTATATTGGATTAA |  |
|  | TTAAAAACCTCAACCTCTAAATTAAATACC | REV |  |  |  |  |
