# Supplementary material for: Novel role for conceptus signals in mRNA expression regulation by DNA methylation in porcine endometrium during early pregnancy
Source: Biol Reprod. 2022 Nov 2;108(1):150–68. doi: 10.1093/biolre/ioac193 (PMC9843678; doi:10.1093/biolre/ioac193)
Supplement: SUPPLEMENTARY_FIGURE_LEGENDS_ioac193 [file supplementary_figure_legends_ioac193.docx]

**SUPPLEMENTARY FIGURE LEGENDS**

**SUPPLEMENTARY FIGURE 1**

Genomic localization of CpG and/or CpN sites within the selected sequences of the analyzed genes.

**SUPPLEMENTARY FIGURE 2**

Uncropped images of Western blot membranes used for representative blots in Figure 1 B, D and F and negative controls with matched normal mouse and rabbit IgG (Suppl. Fig. 2A and C).

**SUPPLEMENTARY FIGURE 3**

Correlation between methylation levels of single CpG/CpN sites localized in sequences under analysis and the expression levels of selected genes in the porcine endometrium treated with E_2__833 ng, E_2__33.3 µg or E_2_+PGE_2_ and in endometrial samples collected from gilts on day 12 of pregnancy or estrous cycle. E_2__833 ng – gilts received infusions of a placebo into one randomly selected horn and E_2_ (833 ng/infusion) into the contralateral horn. E_2__33.3 µg - gilts receiving infusions of a placebo into one randomly selected uterine horn and E_2_ (33.3 µg/infusion) into the contralateral horn. PGE_2_ – gilts receiving infusions of a placebo into one randomly selected horn and PGE_2_ (200 µg/infusion) into the contralateral horn. E_2_+PGE_2_ - gilts receiving infusions of either placebo into one randomly selected horn or E_2_ (33.3 µg/infusion) together with PGE_2_ (200 µg/infusion) into the contralateral horn. AU – arbitrary units.
